# Shen-Hong-Tong-Luo formula ameliorates atherosclerosis by enhancing macrophage efferocytosis through activating the PPARγ/mfge8 pathway

**DOI:** 10.3389/fimmu.2025.1727378

**Published:** 2026-01-20

**Authors:** Yanyu Shi, Zepeng Zhang, Jiaqi Liu, Xiaolin Xu, Xue Xiao, Tianyang Zhang, Yuan Zhang, Haoran Cui, Xiangyan Li, Ying Chen

**Affiliations:** 1 Department of Cardiology, The Affiliated Hospital to Changchun University of Chinese Medicine, Changchun, Jilin, China; 2 Research Center of Traditional Chinese Medicine, The First Affiliated Hospital of Changchun University of Chinese Medicine, Changchun, China; 3 Northeast Asia Research Institute of Traditional Chinese Medicine, Jilin Provincial Key Laboratory of Bio-Macromolecules of Chinese Medicine, Changchun University of Chinese Medicine, Changchun, China; 4 Statistical Center, The Affiliated Hospital to Changchun University of Chinese Medicine, Changchun, China; 5 Department of Endocrinology, The First Affiliated Hospital of Changchun University of Chinese Medicine, Changchun, China

**Keywords:** atherosclerosis, efferocytosis, immunity, macrophage, PPARγ

## Abstract

**Background:**

Atherosclerosis (AS) is a chronic inflammatory disorder driven by dysregulated lipid metabolism and remains a leading cause of cardiovascular morbidity. The Shen−Hong−Tong−Luo (SHTL) preparation has demonstrated clinical benefit in stabilizing atherosclerotic plaques, yet its molecular mechanisms are not fully defined.

**Purpose:**

This research sought to elucidate the protective effects exerted by SHTL on AS progression.

**Methods:**

To investigate the impact of SHTL on macrophage function and plaque stability, we utilized ApoE^-/-^ mice models and bone marrow-derived macrophages (BMDMs) stimulated with lipopolysaccharide (LPS) and oxidized low-density lipoprotein (Ox-LDL). Network pharmacological analysis was conducted to predict potential therapeutic targets of SHTL, with a particular focus on the efferocytosis pathway. These predictions were subsequently confirmed by immunofluorescence (IF) staining and flow cytometry experiments performed on ApoE^-/-^ mice and BMDMs. Furthermore, using data-independent acquisition (DIA) proteomics, milk fat globule-epidermal growth factor 8 (Mfge8) was identified as a critical factor facilitating SHTL-enhanced efferocytosis. Chip-PCR and GW9662 were applied to explore the involvement of peroxisome proliferator-activated receptor gamma (PPARγ) signaling.

**Results:**

SHTL markedly attenuated the progression of AS, demonstrated by reduced plaque formation within both the aortic root and aorta, diminished plasma lipid concentrations, and suppressed inflammatory responses. *In vitro* assays using BMDMs revealed that SHTL significantly inhibited foam cell formation and inflammation induced by Ox-LDL and LPS. Furthermore, SHTL enhanced efferocytosis both *in vivo* and *in vitro* by upregulating Mfge8 expression. Treatment with GW9662 abolished these beneficial effects, confirming that SHTL exerts its protective actions via activation of the PPARγ/Mfge8 pathway.

**Conclusion:**

SHTL demonstrates significant anti-inflammatory and lipid-regulatory effects, attenuating AS progression through the PPARγ/Mfge8 pathway, thereby enhancing macrophage efferocytosis. These findings highlight a novel mechanism by which SHTL may contribute to preventing and treating atherosclerotic diseases.

## Highlights

- Network pharmacology reveals that the anti-AS effect of SHTL is associated with efferocytosis.
- Proteomics shows that SHTL increases the expression of the bridging molecule Mfge8 during efferocytosis.
- SHTL inhibits the progression of AS by targeting the PPARγ/Mfge8 signaling pathway.

## 1 Introduction

AS is characterized by abnormal lipid accumulation in arterial walls, leading to plaque development and potentially causing severe cardiovascular disease. Recent studies have enriched our understanding of AS’s pathophysiological mechanisms, with efferocytosis emerging as a key focus (1, 2). This multistep, intricately controlled process engages both professional phagocytes. It is governed by a collaboration of various signaling molecules. Efferocytosis involves the stages of “find me”, “eat me”, phagocytosis, and post-phagocytic processes, which collectively facilitate the clearance of apoptotic cells (3). This mechanism is critical for maintaining physiological homeostasis and overall health. Efficient efferocytosis removes numerous apoptotic cells within plaques, thereby reducing inflammation and preventing secondary cell necrosis. Conversely, deficient efferocytosis results in the accumulation of apoptotic and necrotic cells within plaques. Substances released from these dead cells exacerbate inflammation and necrosis, causing plaque growth, formation of a necrotic core, and plaque rupture, which aggravate AS (4). Consequently, improving efferocytosis represents a valuable strategy for both preventing and managing AS.

Peroxisome proliferator-activated receptor gamma (PPARγ) functions as a critical transcriptional regulator that coordinately governs lipid metabolism and inflammatory pathways, thereby exerting pleiotropic physiological effects. Emerging evidence demonstrates that PPARγ activation promotes the differentiation of distinct macrophage subsets and potently enhances efferocytosis (5). As a critical “eat me” signaling bridge molecule, milk fat globule-epidermal growth factor 8 (Mfge8) mediates apoptotic cell clearance by establishing a molecular link between phosphatidylserine on apoptotic cell membranes and integrin receptors on phagocytic cells. Genetic deficiency of Mfge8 impairs macrophage-mediated efferocytosis, thereby exacerbating necrotic core formation and plaque instability in atherosclerotic lesions (6). Mechanistically, PPARγ acts as a transcription factor to directly modulate Mfge8 gene expression, thereby augmenting macrophage-mediated apoptotic cell clearance and attenuating the progression of atherosclerosis (7).

Although statins are known to influence efferocytosis via multiple pathways (8), including the inhibition of RhoA prenylation or enhancing peroxisome proliferator-activated receptor and CD36 expression to improve apoptotic cell recognition (9), resistance or intolerance to statins is common. This leads to persistent inflammatory risks, highlighted by elevated C-reactive protein (CRP) levels, potentially precipitating fatal atherosclerotic events (10). Traditional Chinese medicine (TCM) plays a vital role in Chinese healthcare and is a promising source for new drug development (11). Reports have highlighted its role in enhancing efferocytosis, such as the extract from pomegranate peel (12), which prevents MerTK shedding and enhances macrophage efferocytosis. Guanxin Kang (13) promotes apoptotic cell engulfment by upregulating Axl, MerTK, and Tyro3 protein expression, thereby enhancing efferocytosis.

Shen-Hong-Tong-Luo formula (SHTL), developed by the renowned Master of Traditional Chinese Medicine Professor Ren Jixue, is a traditional Chinese herbal prescription designed to ​*tonify Qi* and *resolve blood stasis*, primarily used for treating ​atherosclerosis (14, 15). This formula, which is now an institutional preparation of the Affiliated Hospital of Changchun University of Chinese Medicine (approval No. Z201701D0). Its anti-AS efficacy is mediated through inhibiting arterial plaque formation, aligning with TCM principles of clearing pathogenic stasis and phlegm. Notably, this mechanism parallels modern understanding of macrophage-mediated efferocytosis (apoptotic cell clearance), which prevents necrotic core accumulation in atherosclerotic lesions. Our preliminary studies (16) demonstrate that SHTL significantly attenuates macrophage lipid accumulation—a critical determinant of efferocytotic efficiency. However, the precise molecular mechanisms underlying SHTL-mediated enhancement of macrophage efferocytosis remain incompletely characterized.

In this study, we investigated the anti-AS effects of SHTL using ApoE^-/-^ mice and LPS/Ox-LDL-stimulated BMDMs. Network pharmacology and experimental validation revealed that SHTL attenuates AS by enhancing efferocytosis, with data-independent acquisition (DIA) proteomics identifying Mfge8 as a key regulatory target. Mechanistic studies further demonstrated that SHTL activates the PPARγ pathway, as confirmed by pharmacological inhibition using the PPARγ antagonist GW9662. These experiments potentially yield novel insights into the molecular mechanisms underlying SHTL inhibits AS progression.

## 2 Materials and methods

### 2.1 Preparation of SHTL

The composition of SHTL was depicted in **Supplementary Table S1**, the herbal were procured from Department of Pharmacy at the Affiliated Hospital of Changchun University of Chinese Medicine (Jilin, China), For extraction, the herbal mixture was decocted twice in 300 mL of distilled water at 100°C for 30 min per cycle. The combined aqueous extracts were filtered, centrifuged, and subsequently lyophilized to yield a powdered form for experimental use.

### 2.2 Extraction, differentiation, culture, and identification of BMDMs

BMDMs were isolated from 4 to 6-weeks-old male C57BL/6 mice and cultured in DMEM supplemented with 10% FBS and 20 ng/mL M-CSF (PeproTech) for 7 days. The efficiency of differentiation into F4/80^+^CD11b^+^ macrophages was verified via flow cytometry (BD FACSCanto II) using FITC-anti-F4/80 (clone BM8) and APC-anti-CD11b (clone M1/70) antibodies (BioLegend, 1:200 dilution).

### 2.3 Induction of apoptosis in cells

Jurkat cells were exposed to 254-nm UV lamp for 15 min to induce apoptotic cells. (17) Following irradiation, cells were stained with a dual staining solution of 2 µM Calcein-AM (Thermo, C3100MP) and 1.5 µM propidium iodide (PI, Sigma), diluted 1:1000 in assay buffer. The cell suspension (200 µL) was mixed with 100 µL of staining solution and incubated, immediately followed by flow cytometric analysis.

### 2.4 Preparation and drug administration of the AS mouse model

All experimental mice were purchased from GemPharmatech Co., Ltd. (Nanjing, China) and housed in the specific pathogen-free (SPF)-grade Laboratory Animal Center at Changchun University of Chinese Medicine. The facility maintained regulated environmental conditions: temperature of 20 ± 2°C, relative humidity of 55 ± 10%, and a 12/12-hour light/dark cycle. During both acclimatization and experimental periods, mice had free access to food and water ad libitum. The experiment protocols were reviewed and approved by the Changchun University of Chinese Medicine’s Animal Ethics Committee (Ethics Approval Number 2021099). Animal Experiment 1: ApoE^-/-^ mice (n=50) were allocated into control and various dosage groups (n=10 per group) of model, low (1.4 g/kg), medium (2.8 mg/kg), high (5.6 g/kg) dose SHTL, and rosuvastatin calcium (Rsv, 10mg/kg) after a week of acclimatization. Ten C57BL/6 mice served as untreated controls. All mice, except controls, were fed with high-fat diet whereas controls received standard chow. Treatments were administered daily at 9:00 AM for 10 weeks.

Animal Experiment 2: ApoE^-/-^ mice (n=40) were randomized into model, SHTL (2.8 g/kg oral), GW9662 (1 mg/kg intraperitoneal), (18) and GW9662+SHTL (2.8 g/kg oral + 1 mg/kg intraperitoneal) groups (n=10/group). C57BL/6 mice (n=10) served as blank controls. Diet regimens and vehicle administration were the same as in Experiment 1. SHTL was gavaged daily, and GW9662 was injected intraperitoneally each day. The combined treatment group received both modalities. Treatment lasted 10 weeks, with daily administration at 9:00 AM.

For mouse atherosclerosis study, mice were euthanized by CO_2_ inhalation and perfused with PBS via the left ventricle for 5 minutes. After perfusion, the hearts and aortas were collected and prepared for further analysis.

### 2.5 Gross Oil Red O staining of aortic tissue

The aortas were carefully excised and triple-rinsed in ice-cold PBS (PH 7.4), longitudinally opened, and briefly fixed in 60% isopropanol (v/v). They were then stained with 0.5% Oil Red O (ORO) solution in 60% isopropanol at 37°C. Differentiation was achieved through sequential washes in 60% isopropanol until optimal plaque-lumen contrast was observed.

### 2.6 Tissue sectioning for Oil Red O staining, hematoxylin and eosin staining, and Masson staining

ORO Staining: Cryosections (8μm thickness) were equilibrated to room temperature, and pretreated with 60% isopropanol. Sections were immersed in filtered Oil Red O working solution, then rinsed thrice with distilled water. Unspecific stains were removed by differentiating in 60% isopropanol. The sections were then counterstained with Mayer’s hematoxylin, blued using Scott’s tap water substitute, and mounted in aqueous glycerin gelatin (Beyotime C0187).

H&E Staining: Paraffin-embedded sections (5µm thick) underwent serial deparaffinization and rehydration. After washing in distilled water, sections were stained with hematoxylin, differentiated in 1% acid ethanol, blued in 0.2% ammonia water, and counterstained with eosin. The sections were then dehydrated and cleared before mounting under cover glasses with Neutral balsam (Solarbio G8590).

Masson Staining: Paraffin sections (5μm thick) were dewaxed, rehydrated, and stained in sequence. Nuclear staining utilized Weigert’s iron hematoxylin, followed by differentiation in 1% acid ethanol and bluing in Masson Blue Solution. Cytoplasmic staining employed Ponceau Fuchsin Staining Solution, while collagen was differentiated with 1% phosphomolybdic acid (Solarbio G1340), and fibers were stained with aniline blue. Sections underwent dehydration through an ethanol-xylene series and were mounted in Neutral balsam (Solarbio G8590).

### 2.7 Nile red detection

BMDMs were fixed with 4% paraformaldehyde. After three PBS washes, staining solution (Solarbio G1264) was added for 20 minutes at 25°C in the dark. Fluorescent images were captured using the EVOS M7000.

### 2.8 Determination of inflammatory cytokines and lipid levels

Inflammatory cytokines were quantified using ELISA kits. Serum lipid profiles, including total cholesterol (TC), triglycerides (TG), low-density lipoprotein cholesterol (LDL-C), and high-density lipoprotein cholesterol (HDL-C), were assessed with a fully automated biochemical analyzer.

### 2.9 Quantitative real-time PCR analysis

Total RNA was isolated from macrophages under varying conditions to measure levels of IL-1β, TNF-α, and *Mfge8* using a total RNA kit (Omega, Norcross, GA, USA). One microgram of RNA was reverse transcribed into cDNA with the iScript cDNA synthesis kit (Bio-Rad, USA). Gene expression analyses were conducted using a Bio-Rad CFX96 system, normalizing to β-Actin and quantifying changes via the 2^-ΔΔCt^ method.

IL-1β: Forward primer: TTCAGGCAGGCAGTATCACTC; Reverse primer: GAAGGTCCACGGGAAAGACAC
TNF-α: Forward primer: TTGTCTACTCCCAGGTTCTCT; Reverse primer: GAGGTTGACTTTCTCCTGGTATG
*Mfge8*: Forward primer: CCGCCTCGTCTGTGTATATGG; Reverse primer: CTTGCTATCATAGTTGCTGGCT
β-Actin: Forward primer: CCAGCCTTCCTTCTTGGGTA; Reverse primer: CAAATGCCTGGGTACATGGTG

### 2.10 Network pharmacology and molecular docking

The Traditional Chinese Medicine Systems Pharmacology Database and Analysis Platform (TCMSP) was employed, focusing on oral bioavailability (OB ≥ 30%) and drug-likeness (DL ≥ 0.18). Proteins related to Rhodiola were retrieved from the BATMAN-TCM database. The keyword “AS” facilitated the search for disease-related targets in databases like GeneCards, OMIM, and DisGENET. This search allowed for mapping drug component targets against disease targets, visualizing intersecting genes via a Venn diagram. Networks were constructed in Cytoscape 3.7.2, with further protein-protein interaction (PPI) analysis in the String database and functional enrichment analyses in the DAVID database.

Molecular docking was conducted using AutoDockVina1.1.2 to verify interactions between active SHTL components and key targets. AutoDockVina1.1.2 was employed for molecular docking of active components in SHTL and key targets to verify their interactive activity.

### 2.11 Phagocytosis assay

*In Vitro*: BMDMs were co-cultured with PKH26 Red-labeled apoptotic Jurkat cells (3:1 ratio) for 45 minutes at 37°C. Non-engulfed cells were washed away with ice-cold PBS, and macrophages were labeled with FITC-anti-F4/80 for flow cytometric analysis and imaging post-fixation with 4% PFA (19).

*In Vivo*: Aortic root cryosections (8μm) were fixed and permeabilized and subjected to TUNEL staining (Beyotime C1089). Macrophages were identified with anti-F4/80 antibody (1:100), and nuclei were stained with DAPI (Beyotime C1006). The sections were then mounted with Fluoromount-G™ (Yeasen) and visualized using the EVOS M7000 microscope. Efferocytosis assays at atherosclerotic plaque sites were performed by the immunofluorescence double staining of TUNEL and cleaved F4/80. The phagocytic index was calculated based on the following formula.

Phagocytic index=free apoptotic cells/macrophage associated apoptotic cells.

### 2.12 DIA proteomics and Western-blot

BMDMs were prepared by washing with ice-cold PBS, mechanically detaching using cell scrapers. The resulting cell pellets were immediately frozen in liquid nitrogen and stored at -80°C. DIA proteomic analysis was performed by Novogene Co., Ltd., these pellets were lysed in RIPA and clarified. Protein samples were reduced (10 mM DTT, 56°C), alkylated (50 mM iodoacetamide, 25°C, dark), and digested with trypsin (1:50 w/w, 37°C). The resulting peptides were analyzed using DIA-MS on a Q Exactive HF-X mass spectrometer (Thermo) coupled to an EASY-nLC 1200 system, employing a 60-minute gradient separation on a C18 column. MS settings were: 350–1500 m/z scan range, 30,000 resolution, 45 eV stepped collision energy. Raw data were processed with Spectronaut17 (Biognosys) against the UniProt mouse database. Subsequently, the expression of Mfge8 was assessed by Western blot using a specific primary antibody (Cat. No. 12322, Abclone) following protein separation via 10% SDS-PAGE.

### 2.13 Immunofluorescence staining

Cells cultured in 6-well plates underwent fixation using 4% paraformaldehyde. Subsequent permeabilization involved 0.1% Triton X-100 and blocking with 5% BSA. Cells were then treated with anti-Mfge8 antibody (1:100; PA5-109955) and secondary antibody. Nuclei staining was performed using DAPI, mounting with Fluoromount-G™ (Yeasen), and imaging on an EVOS M7000 microscope.

### 2.14 Immunohistochemistry staining

Paraffin-embedded sections were first deparaffinized in xylene and rehydrated. Antigen retrieval involved heat induction in citrate buffer (pH 6.0, 95°C). Endogenous peroxidase was quenched with 3% H_2_O_2_ (in darkness). Sections were blocked using 3% BSA, followed incubation with the primary antibody. HRP-conjugated secondary antibody was applied. Development of the DAB chromogen was microscopically monitored and stopped with tap water rinse. Counterstaining was done with Mayer’s hematoxylin, differentiated and blued under running water. The sections were then dehydrated and cleared before mounting with Neutral balsam (Solarbio,G8590).

### 2.15 Transfection of *Mfge8* siRNA in cells

BMDMs were transfected with *Mfge8* siRNA (Gene Pharma) using Lipofectamine™ Transfection Reagent (Invitrogen) per manufacturer’s guidelines. A scrambled siRNA served as a control. The sequences for *Mfge8* siRNA were: forward 5′-GGCCUGAAGAAUAACACAAUUTT-3′; antisense 5′-AAUUGUGUUAUUCUUCAGCCTT-3′. BMDMs were seeded in culture plates and maintained at 37°C in a 5% CO_2_ incubator. The transfection mix, prepared by combining 50 nM siRNA and an equivalent volume of Lipofectamine™ 3000, was allowed to incubate for 15 minutes before adding to the plates. Cells were collected after 48 hours for further analysis.

### 2.16 Chromatin immunoprecipitation PCR

The chromatin immunoprecipitation (ChIP) assay was performed using the commercially available Smart-ChIP™ ChIP Kit (Fuming, Shanghai, China) according to the manufacturer’s instructions. Briefly, chromatin was extracted from Raw264.7 cells and subjected to immunoprecipitation using an antibody against PPARγ(Cat No.16643-1-AP, Proteintech). The precipitated DNA was then analyzed by PCR to evaluate the binding of PPARγ to the promoter region of the *Mfge8*gene. The sequences for the *mfge8* promoter was listed below:forward: taggtcttggtgtgcagctg; reverse: cagagcctgaaaaggagggg.

### 2.17 Statistical analysis

Data were presented as means ± standard deviations (SD). Statistical analyses were conducted using GraphPad Prism 8.0. Multiple comparisons were made via one-way ANOVA followed by Tukey’s *post hoc* test, with a significance threshold set at *P* < 0.05.

## 3 Results

### 3.1 SHTL confers protective effects against atherosclerosis in ApoE^-/-^ mice

**Figure 1:**
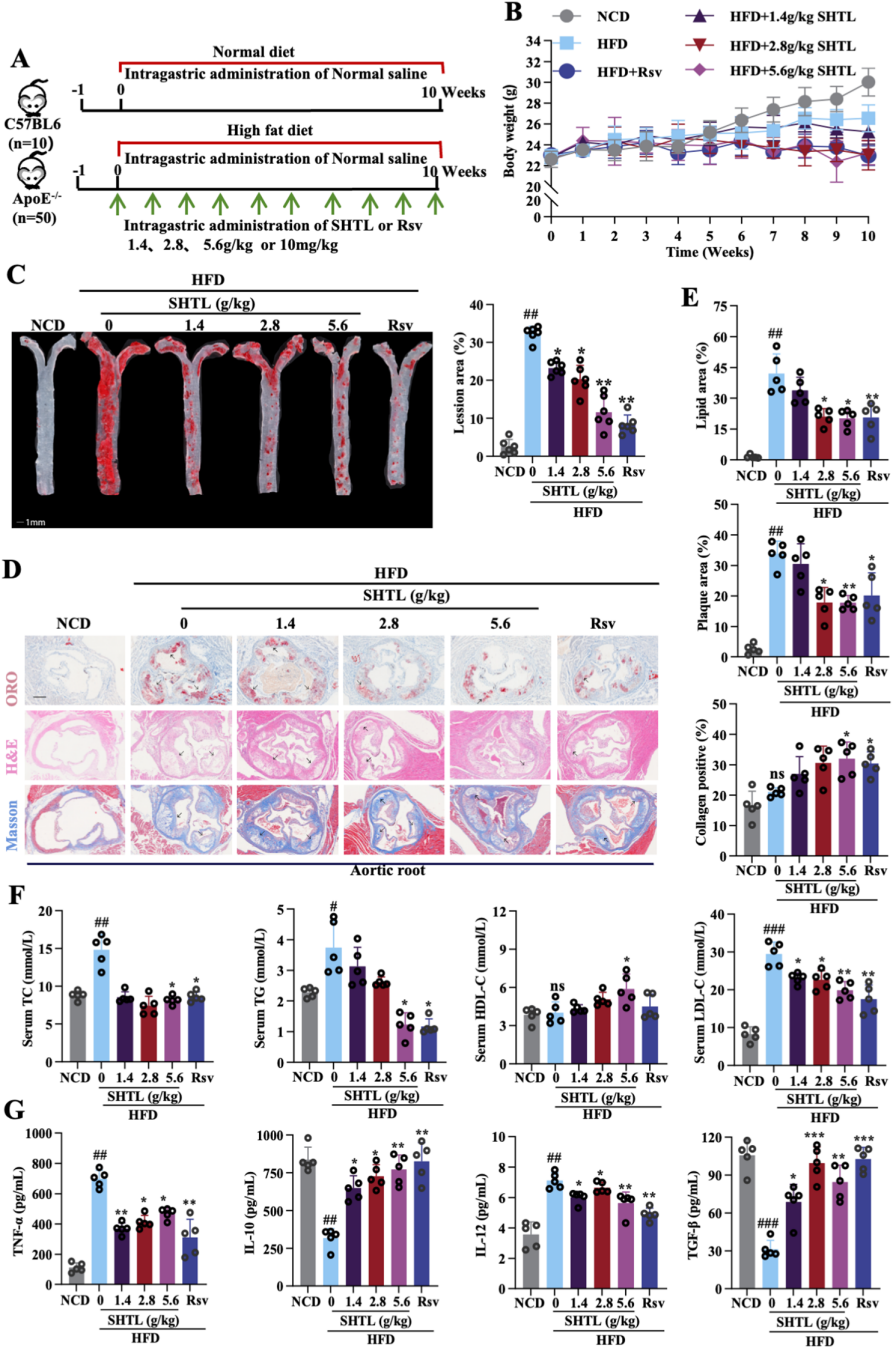
SHTL confers protective effects against atherosclerosis in ApoE^-/-^ Mice. **(A)** Schematic of AS model preparation and drug administration; **(B)** Changes in body weight; **(C)** Results and quantitative analysis of gross Oil Red O staining in aortic tissue;scale bar: 1mm; **(D, E)** Representative images and quantitative analysis results of ORO, H&E, and Masson staining in the aortic root of mice; scale bar: 200μm; **(F)** Levels of TC, TG, LDL, and HDL in mouse serum; **(G)** Changes in TNF-α, TGF-β, IL-10, and IL-12 in mouse serum. ^##^p<0.01 and ^###^p<0.001 indicate significant differences compared with NCD group. *p<0.05, **p<0.01 and ***p<0.001 indicate significant differences compared with the model group. ns indicate not statistically significant.

To assess the influence of SHTL on the progression of AS, we explored its effects on plaque development in ApoE^–/–^ mice. The experimental protocols for AS model creation and SHTL administration are depicted in **Figure 1A**. Throughout the study, all groups exhibited a progressive increase in body weight (**Figure 1B**). Aortic plaque deposition was evaluated using Oil Red O staining, which demonstrated a significant reduction in plaque area in SHTL and Rsv treated groups compared to high-fat diet (HFD)-fed controls (**Figure 1C**). H&E staining of aortic sections revealed typical atherosclerotic lesions in the control group, while SHTL and Rsv treatments notably diminished both plaque burden and necrotic core areas. Further analysis with Masson’s trichrome staining highlighted an enhancement in collagen fiber content within aortic sinus plaques post-treatment (**Figures 1D, E**), underscoring the potential of SHTL in mitigating plaque formation and lipid accumulation. Given the primary role of dysregulated lipid metabolism in arteriosclerosis, we quantified key lipid parameters. Treatments with SHTL and Rsv effectively lowered TC, TG, and LDL levels, while increasing HDL levels (**Figure 1F**). furthermore, we assessed inflammatory markers critical to plaque progression, observing a decrease in TNF-α and IL-12 alongside an increase in TGF-β and IL-10 in treated mice (**Figure 1G**). Collectively, these findings establish that SHTL exerts protective effects against atherosclerotic progression.

### 3.2 SHTL reduces lipid accumulation and inflammatory responses in BMDMs

Macrophages are crucial in forming atherosclerotic plaques, particularly as they transform into foam cells (FCs). To assess the impact of SHTL, BMDMs were harvested from the femurs and tibias, cultured in M-CSF for seven days, and verified for over 90% purity via flow cytometry (**Supplementary Figure S1A**). In an Ox-LDL induced model, Nile Red staining revealed orange-red fluorescence in the cytoplasm of Ox-LDL treated BMDMs, whereas SHTL treatment resulted in a reduced fluorescence intensity (**Supplementary Figure S1C**). ELISA analysis showed dose-dependent reductions in free cholesterol, cholesteryl ester, and TC levels in SHTL-treated BMDMs compared to the Ox-LDL model group (**Supplementary Figure S2A**). In LPS-stimulated BMDMs, SHTL significantly modulated cytokine profiles, reducing pro-inflammatory mediators (TNF-α and IL-12) while increasing anti-inflammatory factors (TGF-β and IL-10) (**Supplementary Figure S2B**). This anti-inflammatory effect was further confirmed at the transcriptional level by downregulation of TNF-α and IL-1β mRNA (**Supplementary Figure S2C**). These findings collectively demonstrate that SHTL exerts dual regulatory effects in macrophages: inhibiting lipid overload and suppressing the inflammatory response, thereby potentially attenuating FCs formation.

### 3.3 SHTL improves AS by promoting the efferocytosis of macrophages

To investigate the mechanistic basis of SHTL in ameliorating AS, network pharmacology approaches were employed. An intersectional analysis of drug targets and AS-associated genes identified 213 overlapping target genes shared between the therapeutic profile of SHTL and atherosclerotic pathways (**Supplementary Figure S3A**). A drug-component-target network, constructed based on degree centrality metrics, identified β-sitosterol, quercetin, kaempferol, luteolin, and stigmasterol as the top five core bioactive components driving potential therapeutic effects (**Supplementary Figure S3B**). Functional enrichment analysis via GO categorized biological annotations into three primary domains: Biological process (BP) were significantly enriched in signal transduction, bidirectional regulation of cell proliferation, apoptotic signaling, and inflammatory response modulation; cellular components (CC) were predominantly localized to subcellular structures such as the nucleus, cytoplasm, and plasma membrane; molecular functions (MF) were characterized by protein-ligand binding and enzyme regulatory activities (**Supplementary Figure S4**). KEGG pathway analysis further demonstrated significant associations with multiple AS-related signaling pathways. Notably, our data unexpectedly revealed that SHTL might be involved in the efferocytosis and PPAR signaling pathway (**Supplementary Figure S3C**).

**Figure 2:**
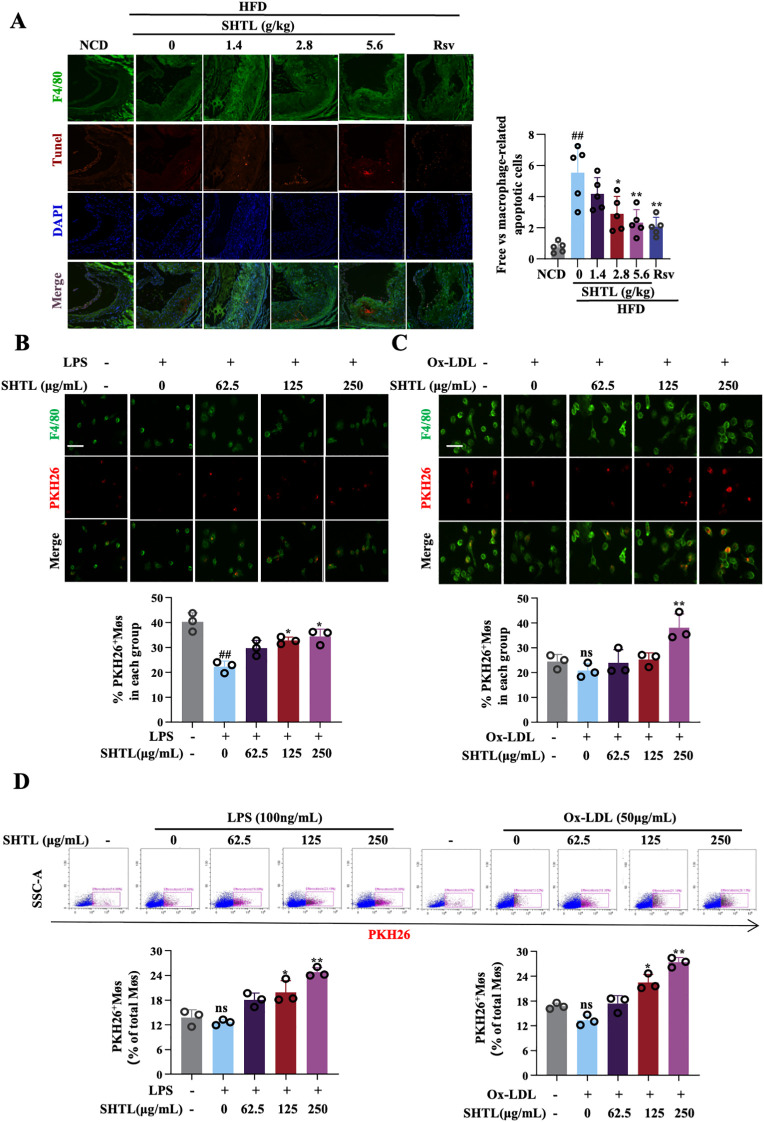
SHTL promotes macrophage efferocytosis in ApoE^-/-^ mice and BMDMs. **(A)** IF detection and quantitative analysis of efferocytosis in ApoE^-/-^ mice;scale bar: 100μm; **(B)** IF detection and quantitative analysis of efferocytosis in the LPS-induced BMDMs;LPS,100ng/mL;scale bar: 100μm; **(C)** IF detection and quantitative analysis of efferocytosis in the Ox-LDL-stimulated BMDMs;Ox-LDL,50μg/mL;scale bar: 100μm; **(D)** Flow cytometry detection and quantitative analysis of efferocytosis in the LPS/Ox-LDL-stimulated BMDMs; ^##^p<0.01 indicate significant differences compared with NCD group. *p<0.05 and **p<0.01 indicate significant differences compared with the model group. ns indicate not statistically significant.

To investigate the role of efferocytosis in the action mechanism of SHTL, *in vivo* studies were performed with ApoE^-^/^-^ mice. IF staining utilized F4/80 to mark macrophages and TUNEL to detect apoptotic cells, evaluating phagocytic function. The phagocytosis index, defined as the proportion of TUNEL^+^ cells engulfed by F4/80^+^ macrophages, was markedly elevated in the model group relative to the blank control, indicating impaired macrophage efferocytosis under atherosclerotic conditions. Treatment with SHTL and Rsv, however, resulted in a notable reduction in the phagocytosis index, suggesting a restoration of macrophage efferocytosis function in the SHTL-treated mice (**Figure 2A**). *In vitro* studies using BMDMs were conducted to evaluate efferocytosis via IF and flow cytometry. Jurkat cell apoptosis exceeded 80%, confirming the stability of the experimental setup (**Supplementary Figure S1B**). IF and flow cytometry analysis revealed that, compared with the LPS-induced and Ox-LDL-stimulated model groups, SHTL treatment at a dose of 250 μg significantly enhanced efferocytosis efficiency in both models (**Figures 2B–D**). However, SHTL did not improve continuous phagocytic capacity when evaluated via the serial phagocytosis assay (**Supplementary Figure S5**). These findings highlight efferocytosis enhancement as a crucial pathway through which SHTL confers protective effects against atherosclerotic progression.

### 3.4 DIA proteomics reveals SHTL attenuates AS through Mfge8-mediated efferocytosis

**Figure 3:**
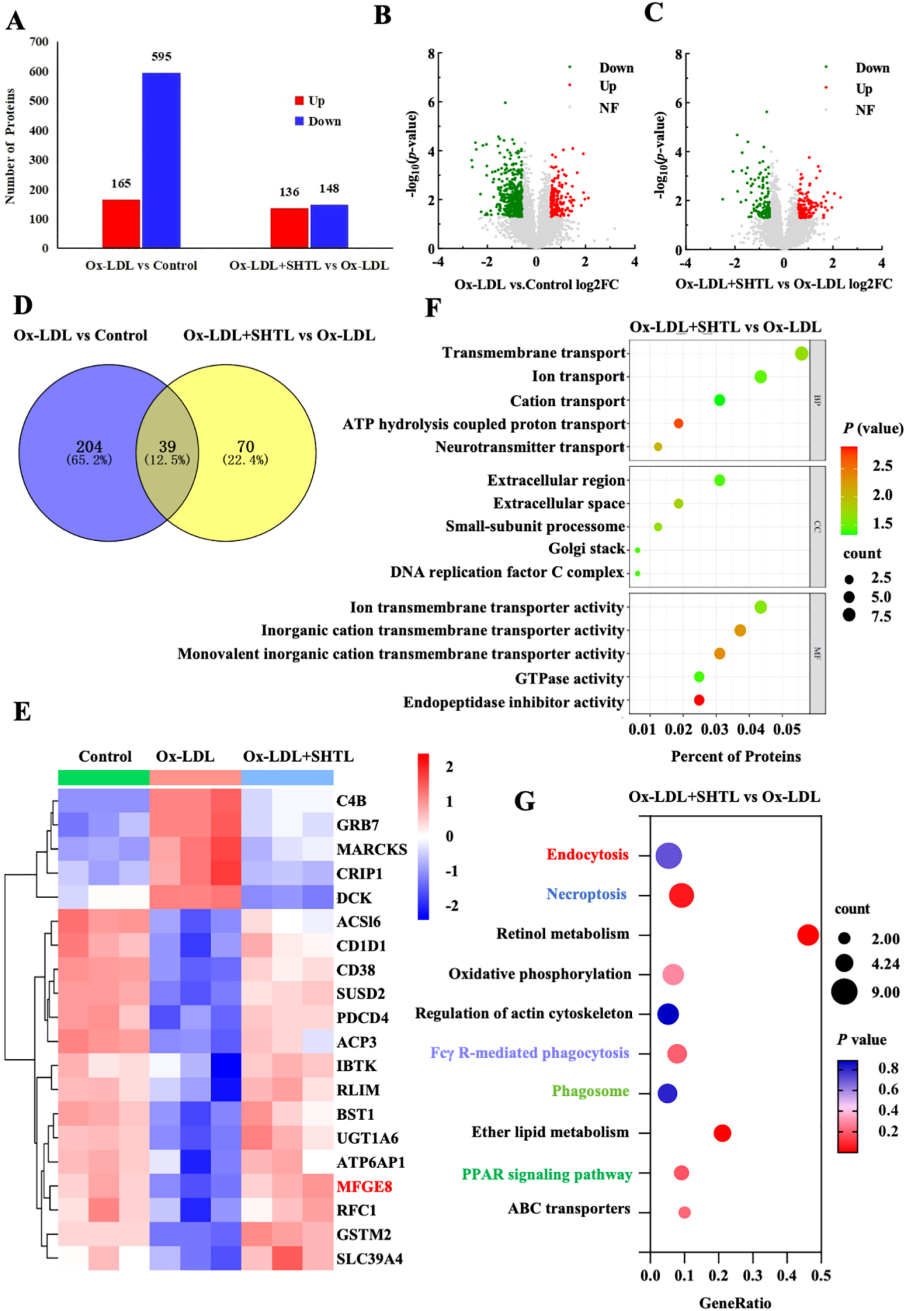
Analysis of differentially expressed proteins after SHTL intervention by DIA proteomics. **(A)** Bar chart of differential proteins; **(B, C)** Volcano plot, green indicates down regulation, red indicates up regulation, and gray indicates no significant change in proteins; **(D)** Venn diagram; **(E)** Heatmap; **(F)** GO enrichment analysis; **(G)** KEGG pathway analysis, Ox-LDL+SHTL *vs* Ox-LDL.

To explore the molecular mechanisms that facilitate the enhancement of efferocytosis by SHTL, an analysis of differential protein expression was conducted utilizing DIA proteomics. A total of 68,270 peptides were identified, corresponding to 6,395 quantifiable proteins across nine samples. Principal component analysis (PCA) revealed distinct differentiation among the three groups, whereas the coefficient of variation (CV) analysis validated the robust reproducibility of the samples (**Supplementary Figures S6A, B**). The analysis of protein expression involved differential screening, applying a fold change threshold of greater than 1.5 (*P* < 0.05) to identify upregulated proteins, while a fold change of less than 0.67 (*P* < 0.05) was utilized for the detection of downregulated proteins. The experimental group displayed 760 differentially expressed proteins, comprising 165 that were upregulated and 595 that were downregulated. The Ox-LDL+SHTL group exhibited 284 differentially expressed proteins, comprising 136 that were upregulated and 148 that were downregulated (**Figure 3A**). Volcano plots visually illustrated the quantity and expression patterns of significant differentially expressed proteins between the groups (**Figures 3B, C**). A Venn diagram illustrated 39 overlapping differentially expressed proteins between the two comparisons (**Figure 3D**), and their expression patterns were organized in a heatmap (**Figure 3E**). The analysis of differentially expressed proteins (DEPs) demonstrated notable enrichment in BP, CC, and MF when comparing the Ox-LDL+SHTL and model groups (**Figure 3F**). KEGG pathway enrichment analysis identified SHTL-mediated regulation of critical pathways—including endocytosis, necroptosis, phagosome, and PPAR signaling pathway—that drive atherosclerotic pathogenesis (**Figure 3G**).

**Figure 4:**
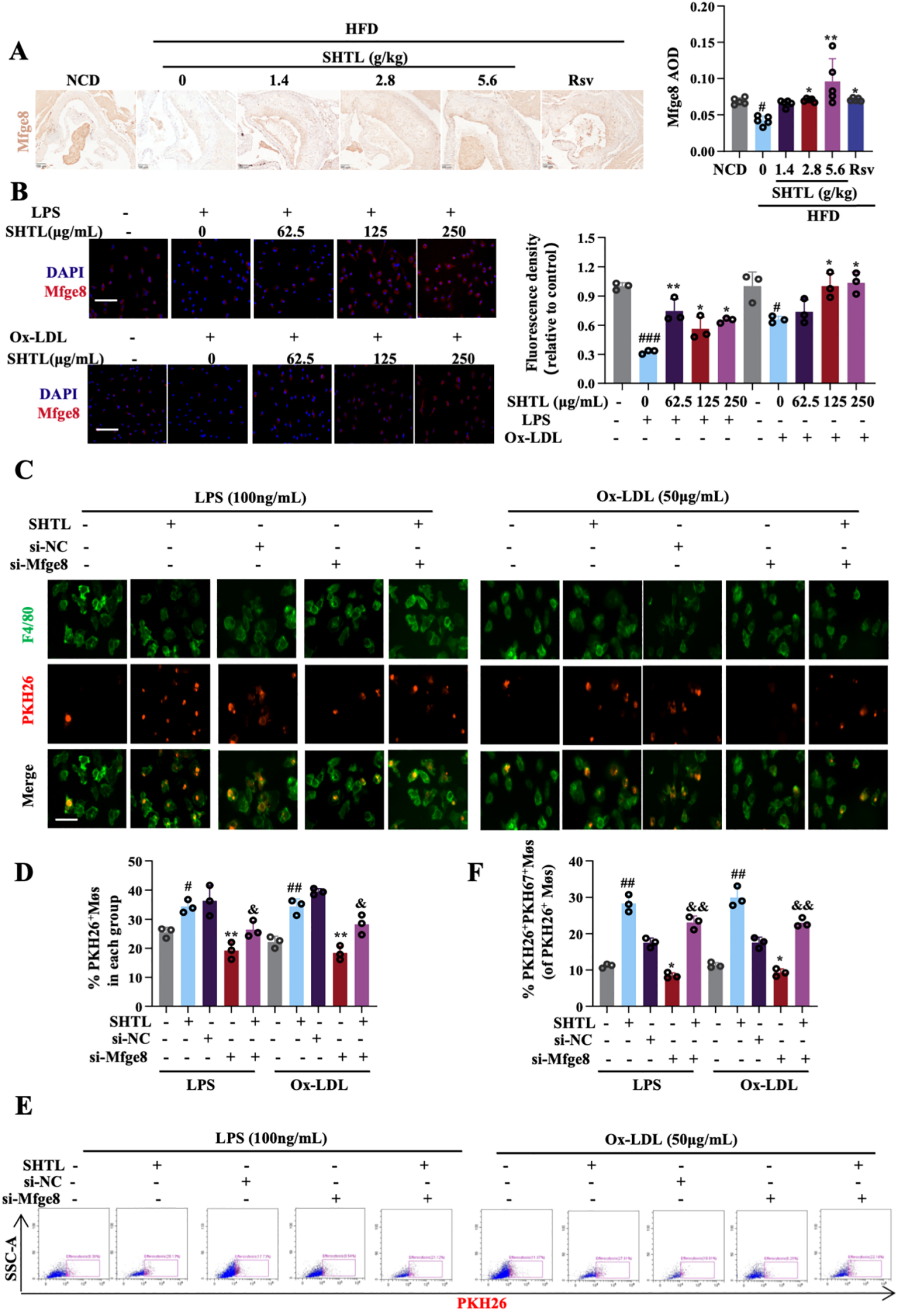
SHTL improves efferocytosis by regulating Mfge8. **(A)** IHC detection and semi-quantitative analysis of Mfge8 expression levels in ApoE^-/-^ mice; scale bar: 100μm; **(B)** IF detection and quantitative analysis of Mfge8 expression levels in LPS/Ox-LDL-stimulated BMDMs;LPS,100ng/mL; Ox-LDL,50μg/mL;scale bar: 100μm; **(C, D)** IF detection and quantitative analysis of phagocytosis ability in the LPS/Ox-LDL-stimulated BMDMs; **(E, F)** Flow cytometry detection and quantitative analysis of phagocytosis ability in the LPS/Ox-LDL-stimulated BMDMs; # indicates comparison with the normal group. * indicates comparison with the model group, ^&^ indicates comparison with the GW9662 group, ^#^p<0.05, ^##^p<0.01and ^###^p<0.001 indicate significant differences compared with NCD group. *p<0.05 and **p<0.01 indicate significant differences compared with the model group. ^&^p<0.05 and ^&&^p<0.01 indicate significant differences compared with the SHTL group.

Intersecting the 39 DEPs with those related to efferocytosis (20), we found that SHTL specifically regulates Mfge8 expression. *In vivo*, IHC results revealed that Mfge8 expression was markedly lower in the model group, while SHTL and Rsv treatments notably restored Mfge8 expression (**Figure 4A**). *In vitro*, experiments showed that SHTL treatment significantly upregulated both Mfge8 protein expression (**Figure 4B**) and gene transcription levels (**Supplementary Figures S7A, B**), suggesting that SHTL promotes efferocytosis by upregulating Mfge8.To validate the role of Mfge8 in SHTL-mediated improvement of efferocytosis, *Mfge8* siRNA3 was selected for experimental knockdown (**Supplementary Figure S7C**). Compared with the si-NC group, the si-*Mfge8*+ LPS/Ox-LDL group exhibited a significant reduction in efferocytosis, demonstrating that downregulation of *Mfge8* inhibits efferocytic activity. However, in the si-*Mfge8*+ LPS/Ox-LDL + SHTL group, efferocytosis was enhanced compared to the si-*Mfge8*+ LPS/Ox-LDL group, although the level remained lower (**Figures 4C–F**). These results indicate that SHTL can partially reverse efferocytic dysfunction induced by Mfge8 deficiency.

### 3.5 SHTL alleviates atherosclerosis via PPARγ signaling pathway

Building on network pharmacology analysis using the KEGG database, we intersected genes involved in the PPAR signaling pathway with those related to efferocytosis and identified three key genes (**Supplementary Figures S8A, B**). Molecular docking revealed strong binding affinities between SHTL’s core components and PPARγ. All binding energies were below -6kJ/mol, indicating stable interactions (**Supplementary Figure S8C**, **Supplementary Table S1**). IHC results demonstrated significantly attenuated PPARγ expression levels in the model group compared to controls, whereas SHTL administration effectively upregulated PPARγ protein expression in treated subjects (**Supplementary Figure S8D**). These findings suggest that SHTL may exert its protective role in AS by modulating the PPARγ signaling pathway.

**Figure 5:**
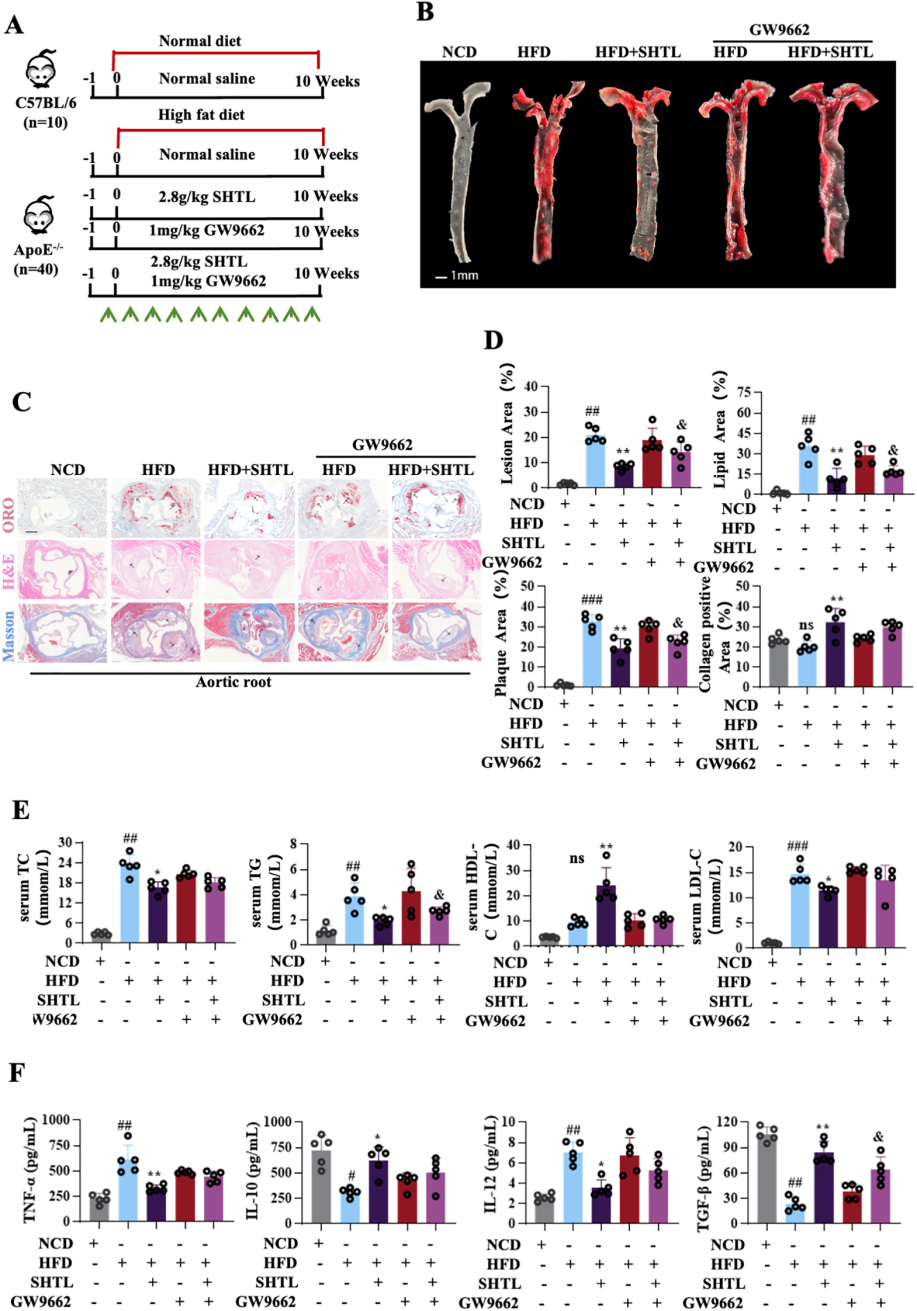
SHTL attenuates atherosclerotic progression *in vivo* via PPARγ. **(A)** Schematic of AS model preparation and drug administration; **(B)** Results of gross Oil Red O staining in aortic tissue; scale bar: 1mm; **(C)** Representative images ORO, H&E, and Masson staining in the aortic root of mice; scale bar: 200μm; **(D)** quantitative analysis of gross Oil Red O staining in aortic tissue and ORO, H&E, and Masson staining in the aortic root of mice; **(E)** Levels of TC, TG, LDL, and HDL in mouse serum; **(F)** Changes in TNF-α, TGFβ, IL-10, and IL-12 in mouse serum; ^#^p<0.05, ^##^p<0.01and ^###^p<0.001 indicate significant differences compared with NCD group. *p<0.05 and **p<0.01 indicate significant differences compared with the model group. ^&^p<0.05 indicate significant differences compared with the SHTL group. ns indicate not statistically significant.

To further investigate this, we employed the PPARγ inhibitor GW9662 for validation purposes. *In vivo*, the methodology for establishing the AS model and administering drug treatment is detailed in **Figure 5A**. In terms of plaque progression, oil red staining results clearly demonstrated a substantial increase in the atherosclerotic plaque area upon PPARγ inhibition. Notably, histopathological analysis further revealed that the suppression of PPARγ signaling pathway significantly exacerbated plaque instability. This was characterized by the expansion of necrotic cores and the depletion of collagen content, both of which are critical indicators of vulnerable plaques (**Figures 5B–D**). Subsequent exploration of the lipid profile in mice uncovered that the inhibition of the PPARγ signaling pathway effectively reversed the lipid-lowering effects of SHTL. Specifically, this led to significant elevations in TC, TG, and LDL-C levels, while concurrently causing a notable decrease in HDL-C levels (**Figure 5E**). Furthermore, we also assessed the levels of serum inflammatory factors in the aorta across the different groups. Results from the ELISA analysis indicated that PPARγ pathway inhibition was associated with a significant upregulation of pro-inflammatory cytokines, including IL-12 and TNF-α. Conversely, the expression levels of anti-inflammatory cytokines, such as IL-10 and TGF-β, were markedly reduced (**Figure 5F**).

*In vitro*, Nile Red staining demonstrated that SHTL treatment led to a significant decrease in lipid-associated orange fluorescence, while the GW9662 group showed a notable increase in fluorescence intensity. Interestingly, the GW9662+SHTL group exhibited a partial reversal of the lipid accumulation that was induced by GW9662 (**Supplementary Figure S9A**). In alignment with these findings, the GW9662+SHTL group demonstrated diminished intracellular cholesterol levels (Free cholesterol, Cholesteryl ester, TC) within macrophages (**Supplementary Figures S9B, C**), suggesting that SHTL mitigates the FCs formation and lipid accumulation induced by GW9662. ELISA profiling revealed that the GW9662+SHTL group inhibited TNF-α, IL-12 while enhancing TGF-β, IL-10 (**Supplementary Figure S9D**). The findings were supported by qPCR analysis, revealing a notable decrease in mRNA levels of TNF-α and IL-1β in the GW9662+SHTL group when compared to the GW9662 group (**Supplementary Figure S9E**). In summary, these results demonstrate that SHTL mediates its anti-atherosclerotic effects via activation of the PPARγ signaling pathway.

### 3.6 SHTL restores efferocytosis via PPARγ-mediated upregulation of Mfge8

**Figure 6:**
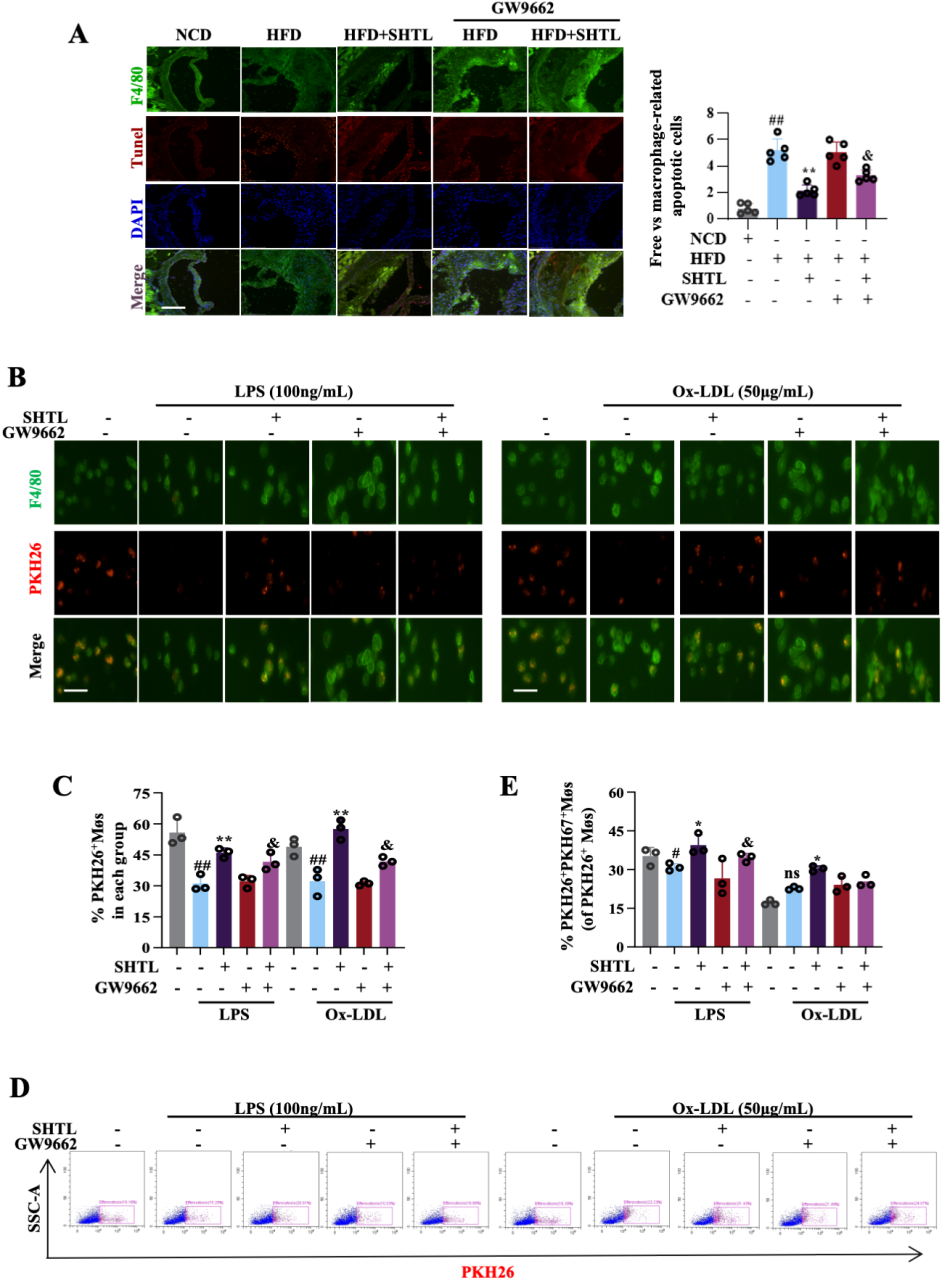
SHTL enhances macrophage efferocytosis via PPARγ. **(A)** IF detection and quantitative analysis of phagocytosis ability in ApoE^-/-^ mice; scale bar: 100μm; **(B, C)** IF detection and quantitative analysis of phagocytosis ability in the LPS/Ox-LDL-stimulated BMDMs;scale bar: 100μm; **(D, E)** Flow cytometry detection and quantitative analysis of phagocytosis ability in the LPS/Ox-LDL-stimulated BMDMs; ^#^p<0.05, and ^##^p<0.01 indicate significant differences compared with NCD group. *p<0.05 and **p<0.01 indicate significant differences compared with the model group. ^&^p<0.05 indicate significant differences compared with the SHTL group. ns indicate not statistically significant.

Previous studies have implicated PPARγ in the regulation of efferocytosis. In line with these findings, our *in vivo* experiments demonstrated that SHTL mitigated the GW9662-induced increase in the phagocytosis index (**Figure 6A**). *In vitro*, efferocytosis efficiency, assessed by IF and flow cytometry, was enhanced in SHTL-treated macrophages but impaired by GW9662. The GW9662+SHTL group showed restored efferocytic activity, surpassing that of the GW9662 group (**Figures 6B–D**). Similarly, the sustained phagocytic capacity of macrophages was not improved (**Supplementary Figure S10**). These findings collectively indicate that SHTL exerts anti-AS effects by restoring PPARγ-dependent efferocytosis.

**Figure 7:**
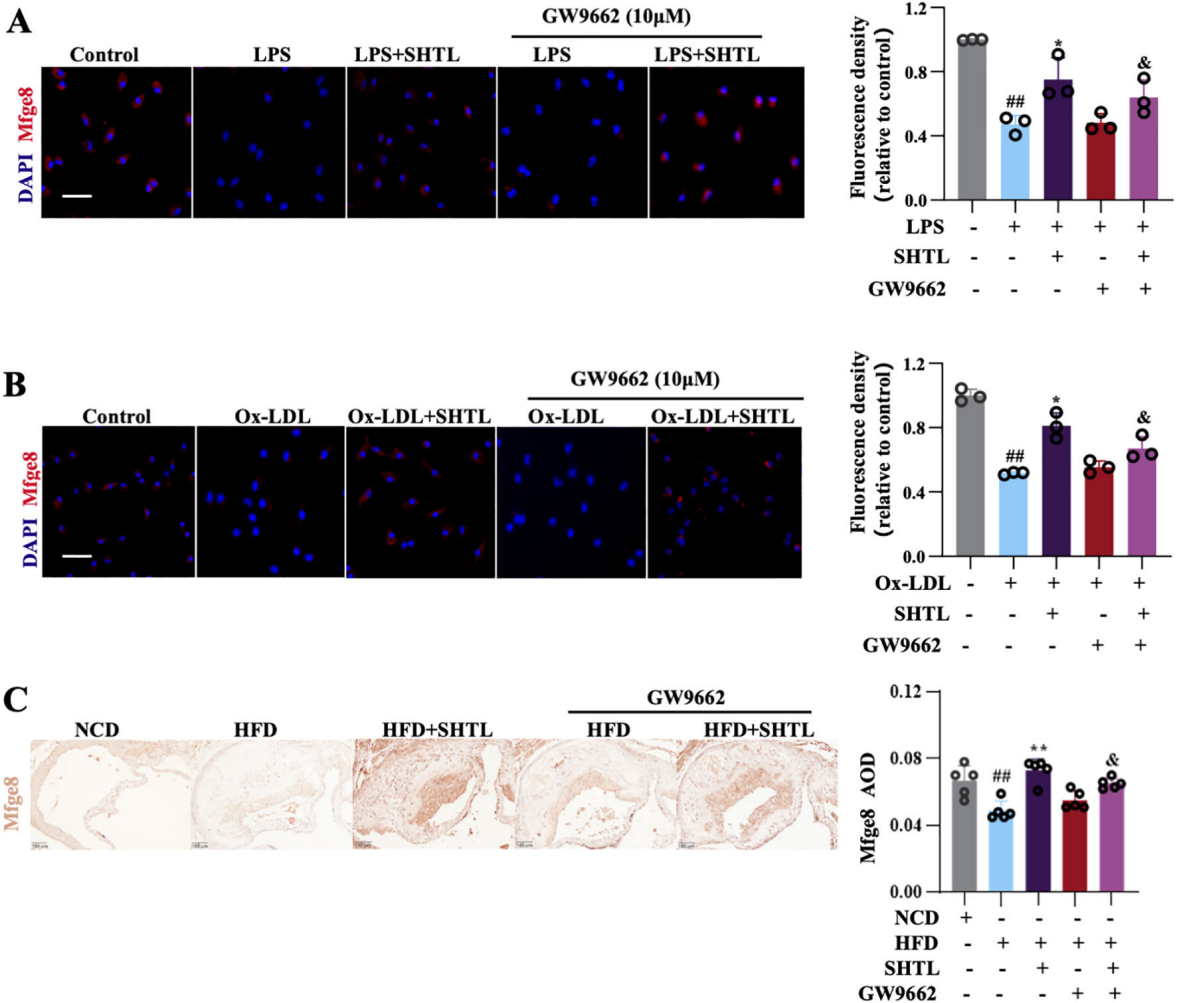
SHTL upregulates Mfge8 through PPARγ. **(A)** IF detection and quantitative analysis of Mfge8 expression levels in LPS-induced BMDMs;scale bar: 100μm; **(B)** IF detection and quantitative analysis of Mfge8 expression levels in Ox-LDL-induced BMDMs;scale bar: 100μm; **(C)** IHC detection and semi-quantitative analysis of Mfge8 expression levels in ApoE^-/-^ mice;scale bar: 100μm; # indicates comparison with the normal group. ^##^p<0.01 indicate significant differences compared with NCD group. *p<0.05 and **p<0.01 indicate significant differences compared with the model group. ^&^p<0.05 indicate significant differences compared with the SHTL group. ns indicate not statistically significant.

Based on these findings, we hypothesized a positive regulatory interaction between PPARγ and Mfge8. To test this, we conducted experimental studies utilizing the PPARγ inhibitor GW9662. *In vitro*, IF analysis demonstrated elevated levels of Mfge8 protein in the SHTL group, diminished levels in the GW9662 group, and a recovery of Mfge8 expression in the GW9662+SHTL group (**Figures 7A, B**). *In vivo*, IHC results demonstrated that the GW9662+SHTL group exhibited a notable elevation in Mfge8 protein expression (**Figure 7C**). The gathered evidence indicates that PPARγ plays a crucial role in promoting macrophage efferocytosis through the transcriptional upregulation of Mfge8, thereby supporting the anti-AS properties of SHTL.

## 4 Discussion

**Figure 8:**
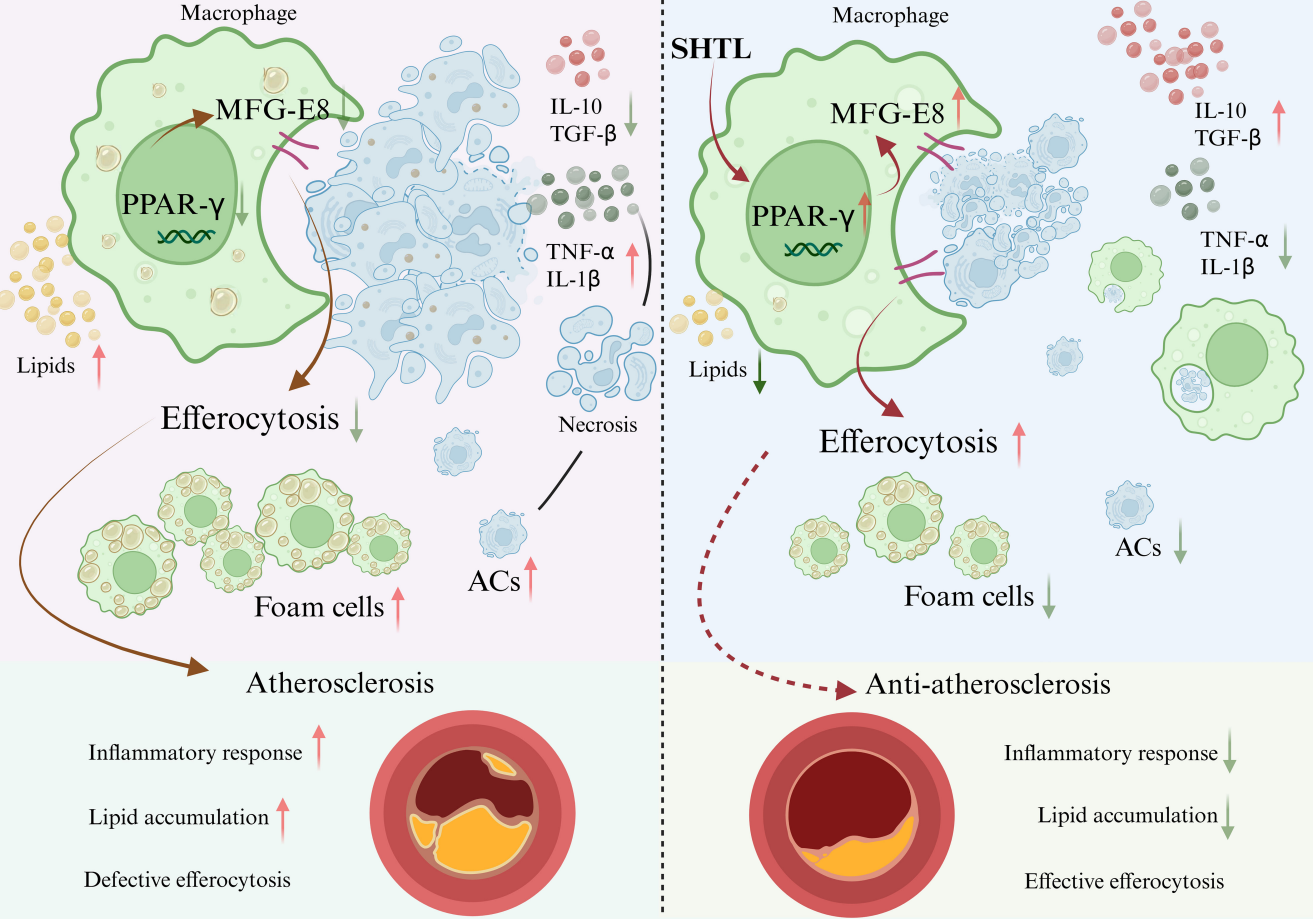
SHTL alleviates atherosclerosis by enhancing macrophage efferocytosis via the PPARγ/mfge8 Pathway Left panel (Untreated): Deficient efferocytosis leads to accumulation of ACs, driving foam cell formation, persistent inflammation, and necrotic core expansion, which collectively promote atherosclerotic plaque progression. Right panel (SHTL-treated): SHTL activates PPARγ, which upregulates MFG-E8. This increase in MFG-E8 enhances macrophage efferocytosis, leading to efficient clearance of ACs. The consequent removal of ACs suppresses foam cell formation, promotes an anti-inflammatory cytokine profile (e.g., IL-10, TGF-β), and inhibits pro-inflammatory mediators (e.g., TNF-α, IL-1β). Together, these effects reduce inflammation and lipid accumulation, thereby promoting plaque stabilization and regression.

The impaired phagocytic function of macrophages is a key factor in the accumulation of apoptotic cells under the endothelium, directly accelerating the progression of atherosclerosis (21). Currently, there are few targeted treatment strategies available (22). This study found that SHTL could inhibit the AS process of ApoE^-/-^ mice by improving the defective efferocytosis of macrophages. Notably, proteomic analysis indicated that SHTL can significantly upregulate the expression of bridging factor Mfge8, which is necessary to the phagocytic function of macrophages. We further applied GW9662 found that PPARγ is the key upstream factor in SHTL upregulating Mfge8 to improve efferocytosis in BMDMs and ApoE^-/-^ mice, which demonstrates a novel mechanism of SHTL improving AS (**Figure 8**).

Growing evidence suggests that macrophages, which play crucial roles in regulating inflammation, phagocytosis of lipids, and the clearance of apoptotic cells, are key targets in the treatment of AS (23). The excessive accumulation of lipids is a primary cause of macrophage foam cell formation and subsequent cellular damage (24). Our study demonstrated that SHTL significantly attenuated Ox-LDL-induced lipid accumulation in BMDMs, as evidenced by reduced levels of free cholesterol, CE, and TC. Furthermore, in LPS-induced inflammatory models, SHTL effectively suppressed the expression of pro-inflammatory mediators including TNF-α, IL-12, and IL-1β, while concurrently enhancing the production of immunomodulatory cytokines TNF and IL-10. Efferocytosis, the process by which phagocytic cells engulf and eliminate apoptotic cells, is a vital mechanism that prevents the accumulation of these cells in plaques and the onset of secondary necrosis (25). In the early stages of AS, macrophages are pivotal in efferocytosis, effectively clearing apoptotic cells from the intimal layer and thereby limiting lesion progression (26). However, in advanced AS, macrophage phagocytic function declines, impairing their ability to clear apoptotic cells efficiently. This failure to clear apoptotic cells triggers a strong inflammatory response, which disrupts cholesterol reverse transport and increases plaque vulnerability (27). Furthermore, reduced clearance of apoptotic cells leads to enhanced secondary necrosis, initiating a self-perpetuating cycle that accelerates disease progression (28, 29). Our findings suggest that SHTL can reduce macrophage foam cell formation and inflammation, thereby inhibiting macrophage apoptosis—a key pathological factor contributing to AS. Additionally, SHTL helps maintain macrophage homeostasis by stabilizing cell functions. We also observed that SHTL enhances macrophage phagocytosis in both Ox-LDL and LPS-induced BMDMs, improving macrophage phagocytic efficiency in the mouse aortic sinus. Collectively, these results demonstrate that SHTL inhibits the progressive development of AS plaques by promoting macrophage phagocytosis.

Macrophage phagocytosis involves a complex process that includes the activation of phagocytic receptors, bridging factors, and recognition mechanisms (30, 31). Using DIA proteomics, we identified that SHTL regulates 284 DEPs, which are significantly enriched in pathways related to programmed cell death, phagosomes, and FcγR-mediated phagocytosis. Further analysis of 39 DEPs associated with efferocytosis revealed that Mfge8 may be a potential target of SHTL. Mfge8, a secretory glycoprotein containing a lactadherin domain, plays a critical role in intercellular signaling and extracellular matrix remodeling (32). As an essential component of the efferocytosis process, Mfge8 is present on the membranes of phagocytic cells and interacts with “eat me” signal molecules on apoptotic cells, including αvβ3 integrin receptors and scavenger receptors, thereby facilitating the removal of apoptotic cells (33, 34). Our results showed that SHTL can upregulate the expression of Mfge8 in BMDMs and the aortic sinus. Furthermore, silencing *Mfge8* using siRNA attenuated the enhancement effect of SHTL on OxLDL and LPS-induced macrophage phagocytosis to varying degrees, confirming that Mfge8 is a key target through which SHTL improves macrophage phagocytosis.

AS involves disruptions in lipid metabolism and inflammation, with PPAR-γ playing a central role in linking these processes (35, 36). Upon activation, PPAR-γ regulates lipid metabolism in macrophages by forming a heterodimer with RXR and binding to PPREs, reducing lipid accumulation and modulating lipid deposition. PPAR-γ also stabilizes AS plaques by suppressing inflammation and promoting cholesterol reverse transport (37, 38). In our study, treatment with the PPAR-γ inhibitor GW9662 increased inflammation, lipid accumulation, and plaque instability. However, the GW9662+SHTL group showed reduced inflammation, improved lipid deposition, and greater plaque stability, suggesting that SHTL targets PPAR-γ to exert anti-AS effects. PPAR-γ also induces the expression of scavenger receptors (e.g., CD36, SR-A) in macrophages, which are vital for phagocytosis (39). Additionally, PPAR-γ promotes efferocytosis by modulating inflammatory responses (40). In our experiments, efferocytosis was reduced in the GW9662 group but enhanced in the GW9662+SHTL group, indicating that SHTL improves macrophage function. Moreover, PPAR-γ activation upregulates Mfge8 expression, which facilitates apoptotic cell clearance (41). Our ChIP-qPCR analysis provided direct evidence that PPARγ binds to the promoter of Mfge8, identifying it as a key transcriptional regulator of this gene. This result solidifies the missing mechanistic link in our proposed pathway. Consistent with this, the observed reduction in Mfge8 expression with GW9662 and its rescue by SHTL co-treatment strongly supports the model that SHTL enhances efferocytosis and slows AS progression by activating the PPARγ-Mfge8 axis. The partial restoration of efferocytosis following Mfge8knockdown, however, indicates that SHTL’s effects are not exclusively mediated by this pathway, hinting at the involvement of parallel compensatory mechanisms.

## 5 Conclusions

In conclusion, our research underscores the notable therapeutic promise of SHTL in addressing AS. Network pharmacology analyses indicate that SHTL influences AS by promoting the process of efferocytosis. Further examination utilizing DIA proteomics revealed Mfge8, an essential mediator in efferocytosis, as a significant target modulated by SHTL. Our results indicate that SHTL diminishes lipid accumulation and inflammation in macrophages through the activation of PPARγ, while maintaining the phagocytic capabilities of these immune cells. Furthermore, the application of the PPARγ inhibitor GW9662 allowed us to validate that the PPARγ/Mfge8 pathway is crucial in the mechanism through which SHTL promotes efferocytosis, thus reducing the advancement of AS.

## Data Availability

All relevant data is contained within the article: The original contributions presented in the study are included in the article/**Supplementary Material**, further inquiries can be directed to the corresponding authors.
