# Supplementary material for: Shen-Hong-Tong-Luo formula ameliorates atherosclerosis by enhancing macrophage efferocytosis through activating the PPARγ/mfge8 pathway
Source: Front Immunol. 2026 Jan 20;16:1727378. doi: 10.3389/fimmu.2025.1727378 (PMC12864095; doi:10.3389/fimmu.2025.1727378)
Supplement: Supplementary file 2 [file DataSheet2.pdf]

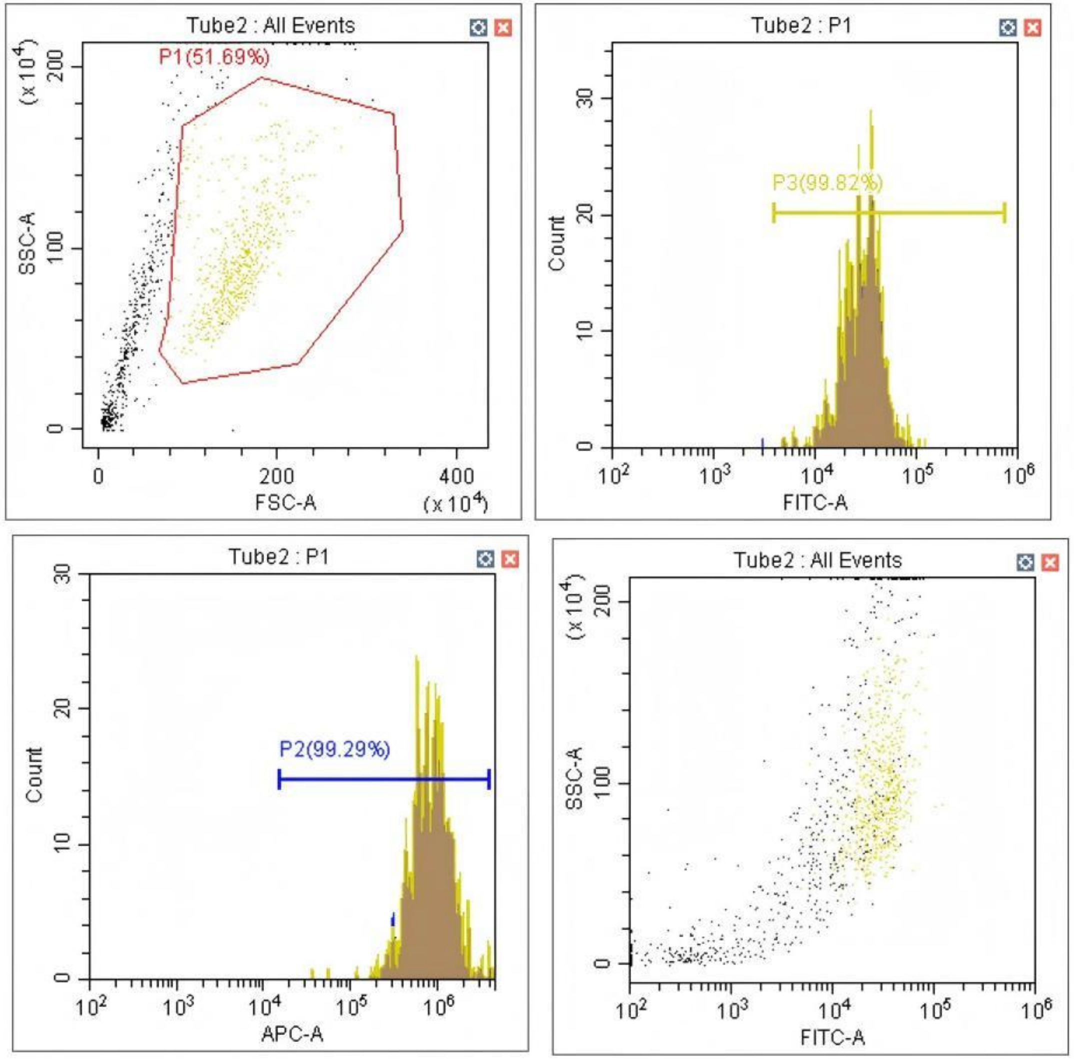

Tube Name: Tube2

Sample ID:

Population	Events	% T...	% P...	SD FITC-A	CV APC-A	SD APC-A	CV FITC-A	Mean FITC-A	Mean APC-A
All Events	1095	100....	100....	17321.6	101.32%	678368.9	79.30%	21842.4	669555.5
P1	566	51.6...	51.6...	13646.3	65.73%	622630.3	44.10%	30940.6	947247.9
P2	562	51.3...	99.2...	13674.1	56.78%	522020.1	44.26%	30892.7	919374.4
P3	565	51.6...	99.8...	13607.3	65.65%	622582.8	43.91%	30990.2	948395.3

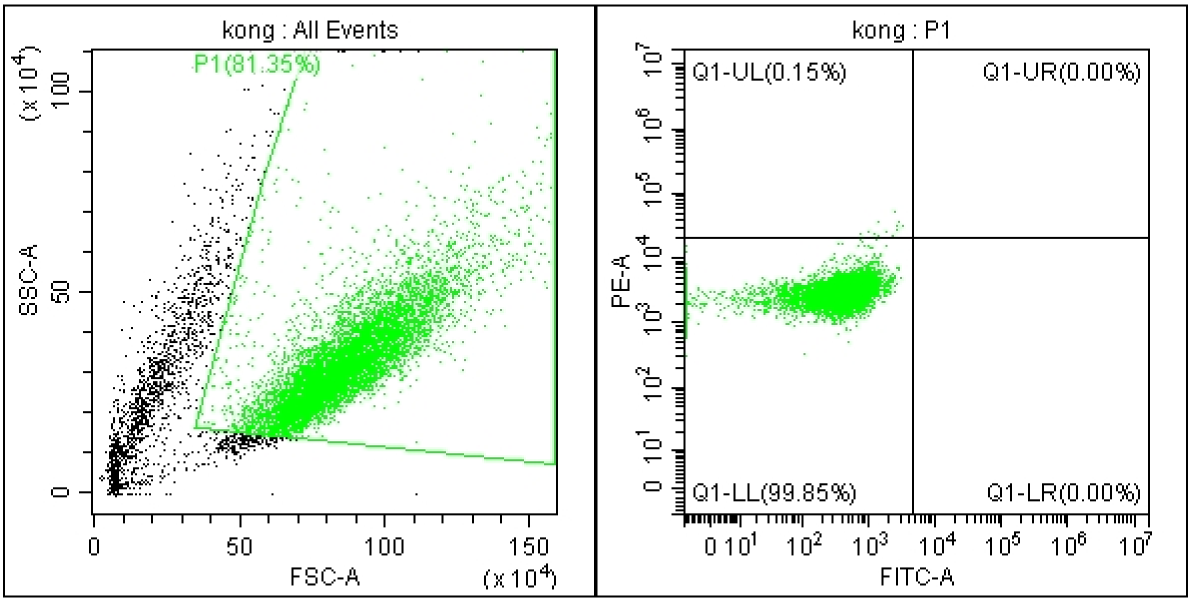

试管名称： kong

样本ID：

群体	颗粒数	%总数	%父群
All Events	10000	100.00%	100.00%
P1	8135	81.35%	81.35%
Q1-UR	0	0.00%	0.00%
Q1-UL	12	0.12%	0.15%
Q1-LL	8123	81.23%	99.85%
Q1-LR	0	0.00%	0.00%

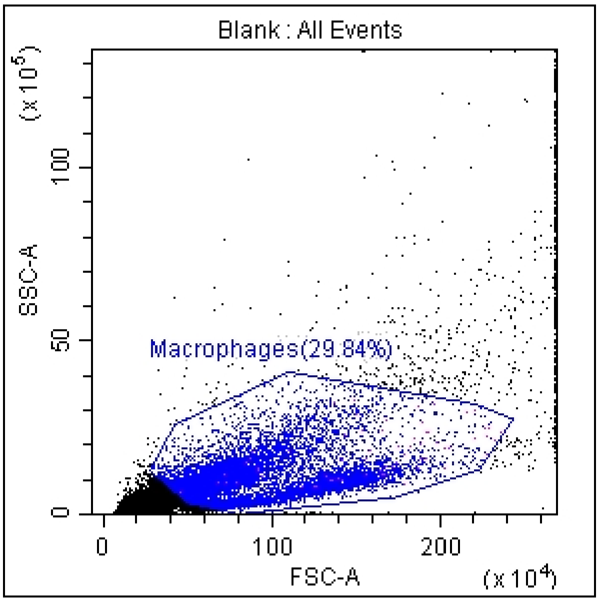

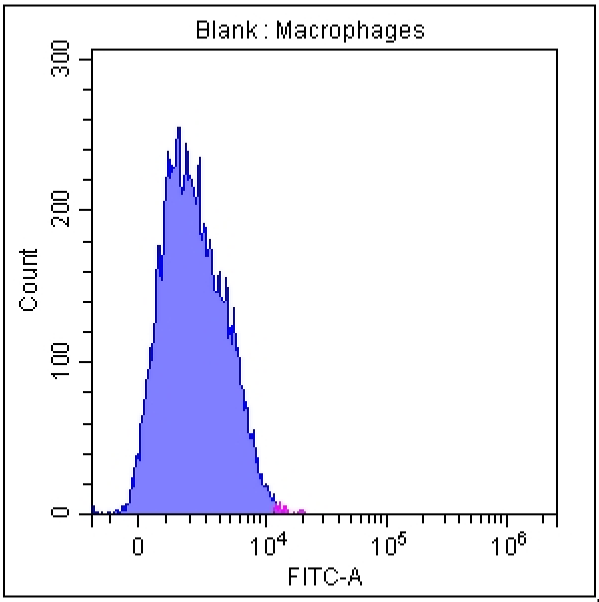

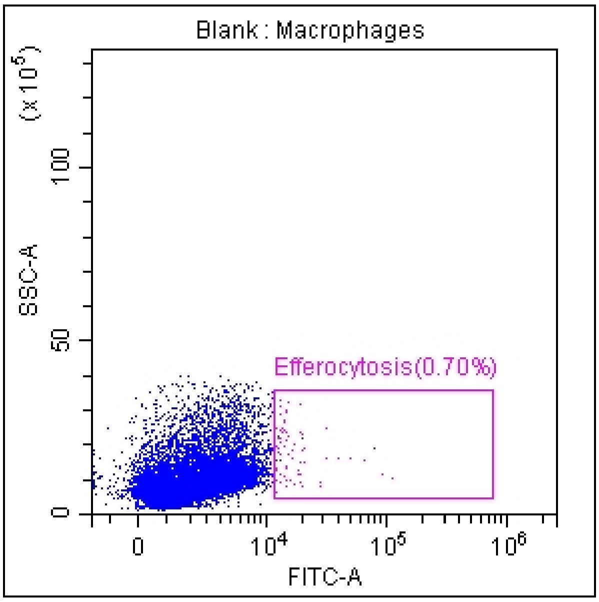

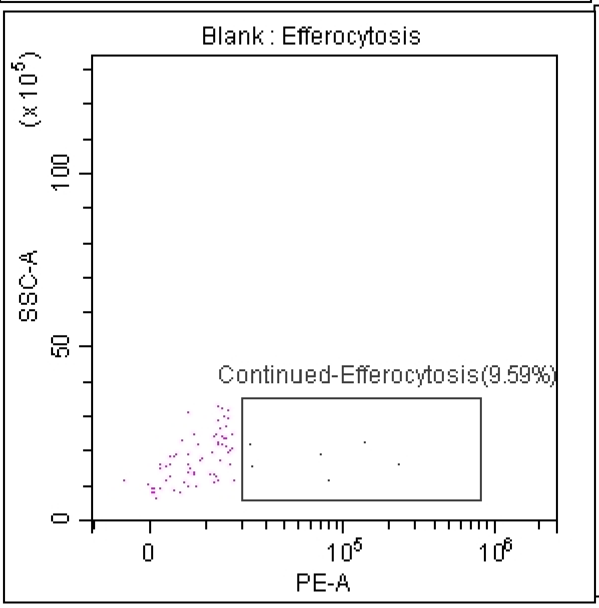

试管名称: Blank

样本ID:

群体
All Events
Macrophages
Efferocytosis
Continued-Efferocytos

	颗粒数	%总数	%父群
	34751	100.00%	100.00%
	10368	29.84%	29.84%
	73	0.21%	0.70%
sis	7	0.02%	9.59%
