# Supplementary figures and images for: Shen-Hong-Tong-Luo formula ameliorates atherosclerosis by enhancing macrophage efferocytosis through activating the PPARγ/mfge8 pathway
Source: Front Immunol. 2026 Jan 20;16:1727378. doi: 10.3389/fimmu.2025.1727378 (PMC12864095; doi:10.3389/fimmu.2025.1727378)

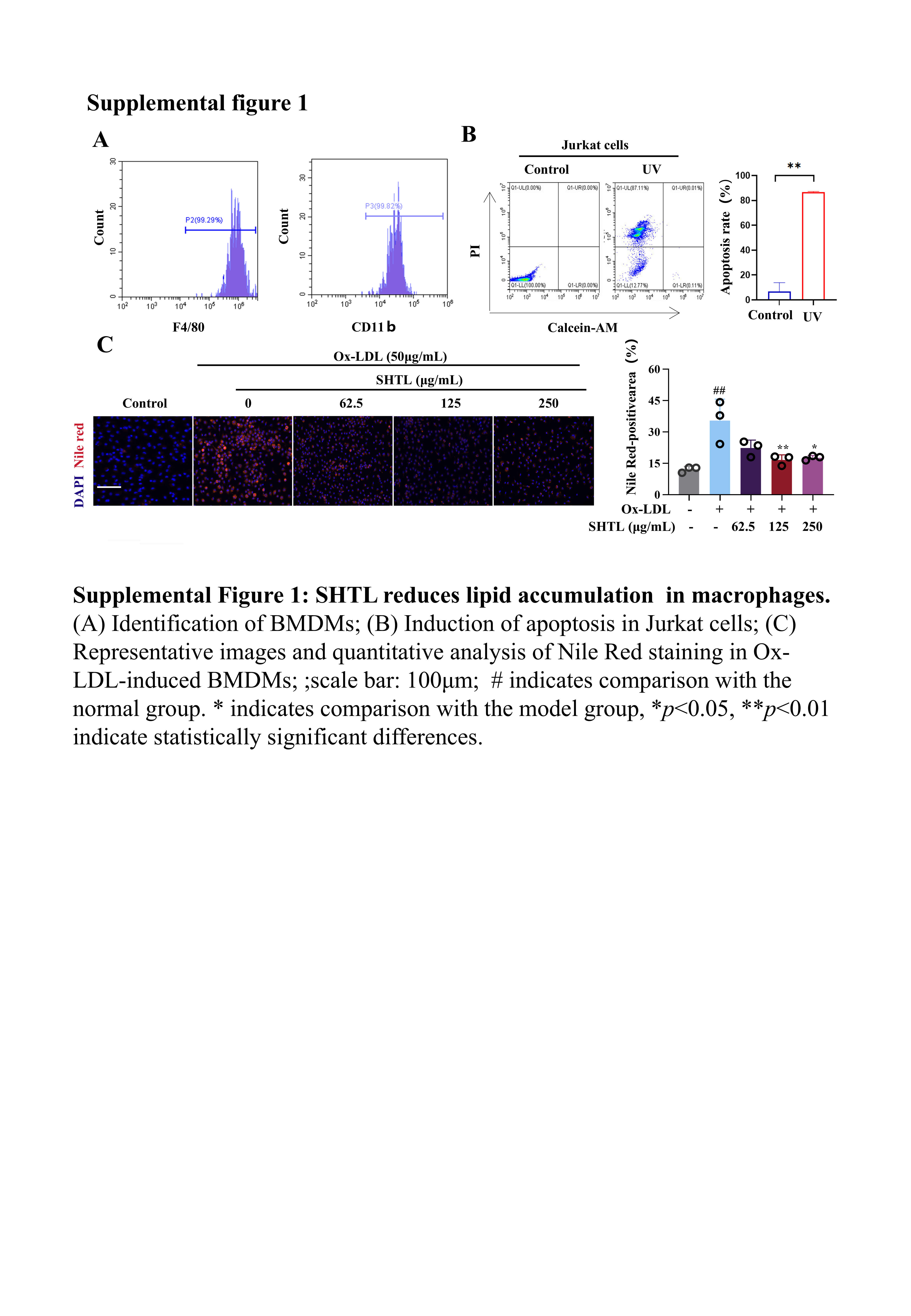

Supplement: Supplementary file 3 [file Image1.tif]

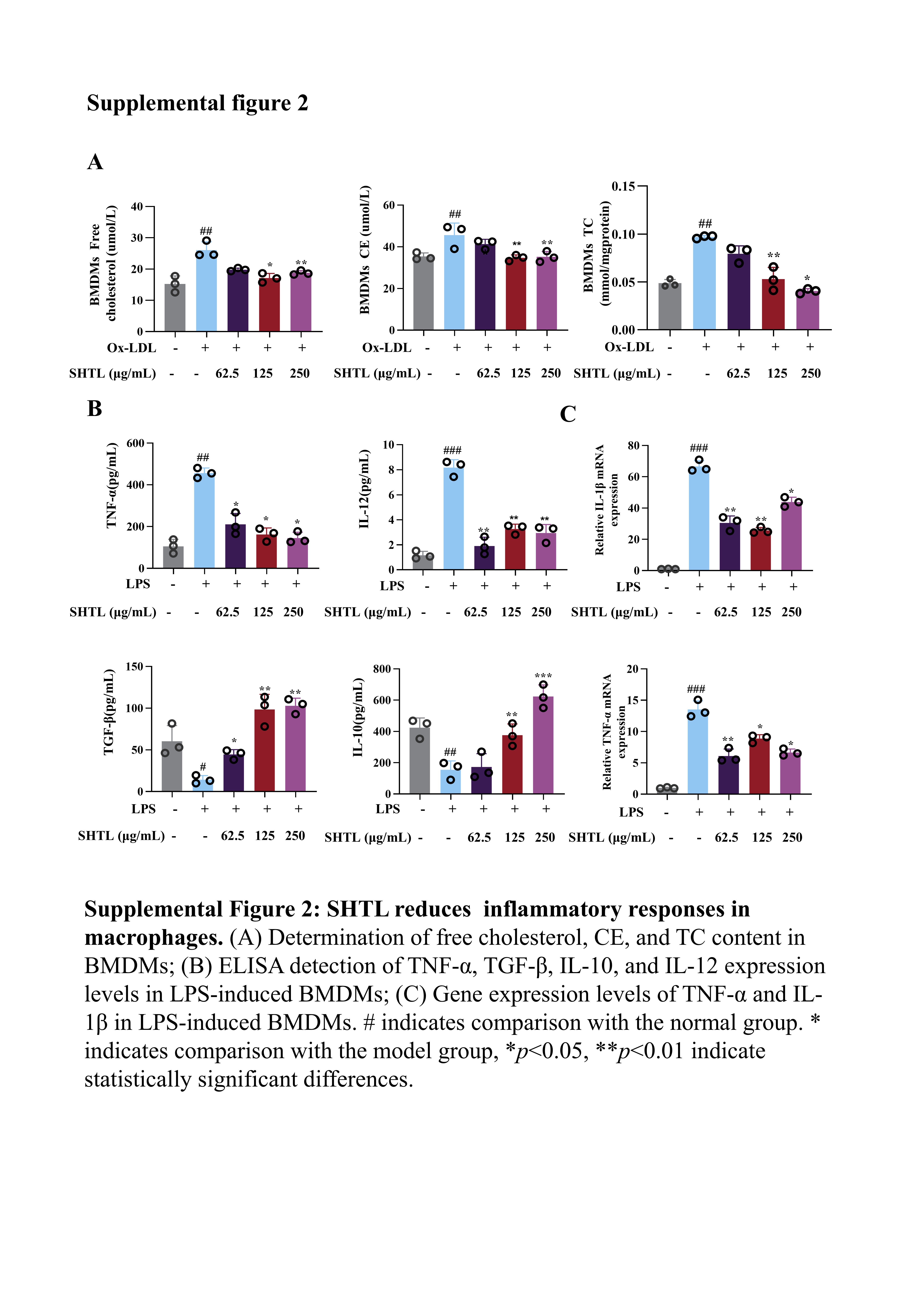

Supplement: Supplementary file 4 [file Image2.tif]

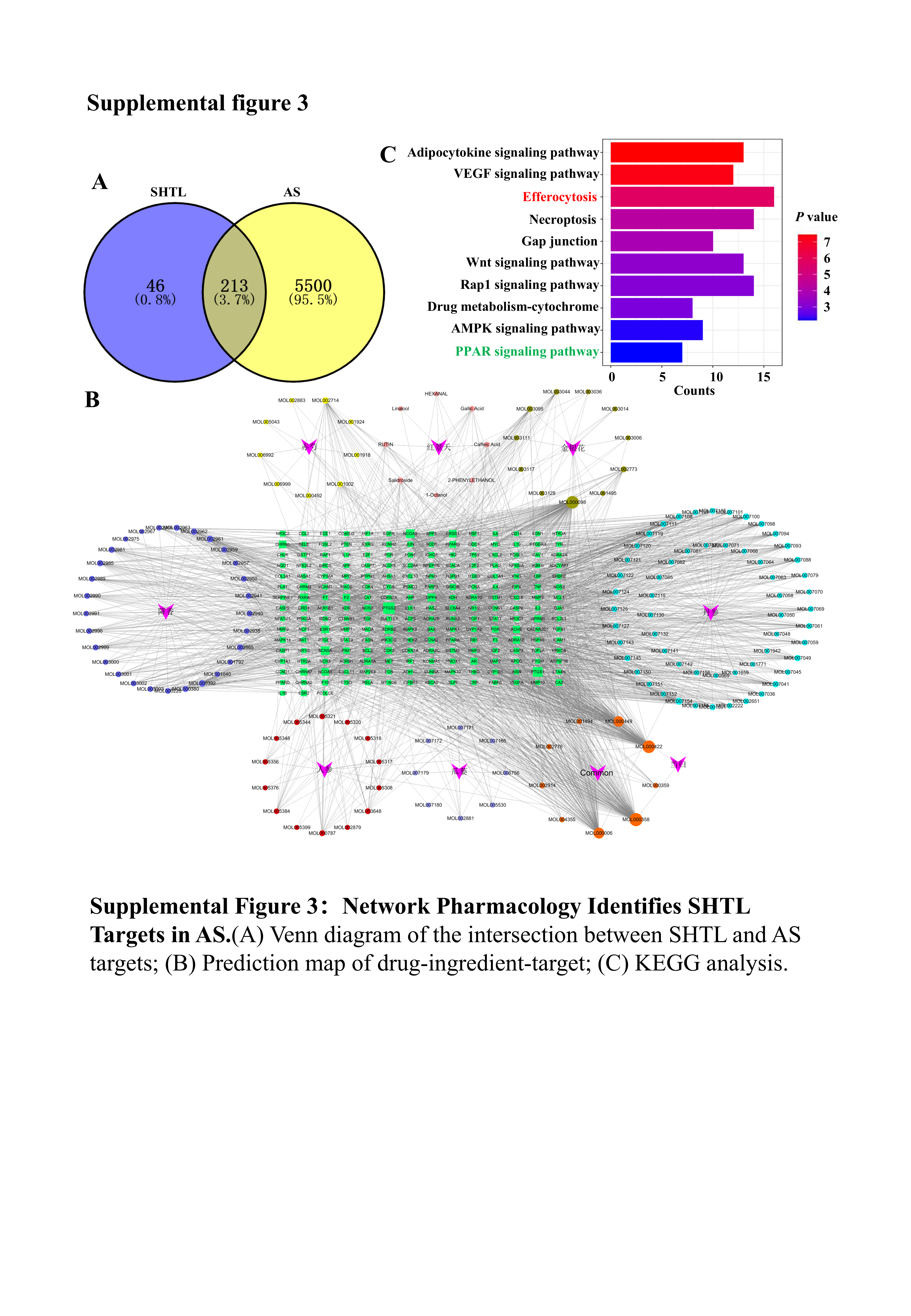

Supplement: Supplementary file 5 [file Image3.tif]

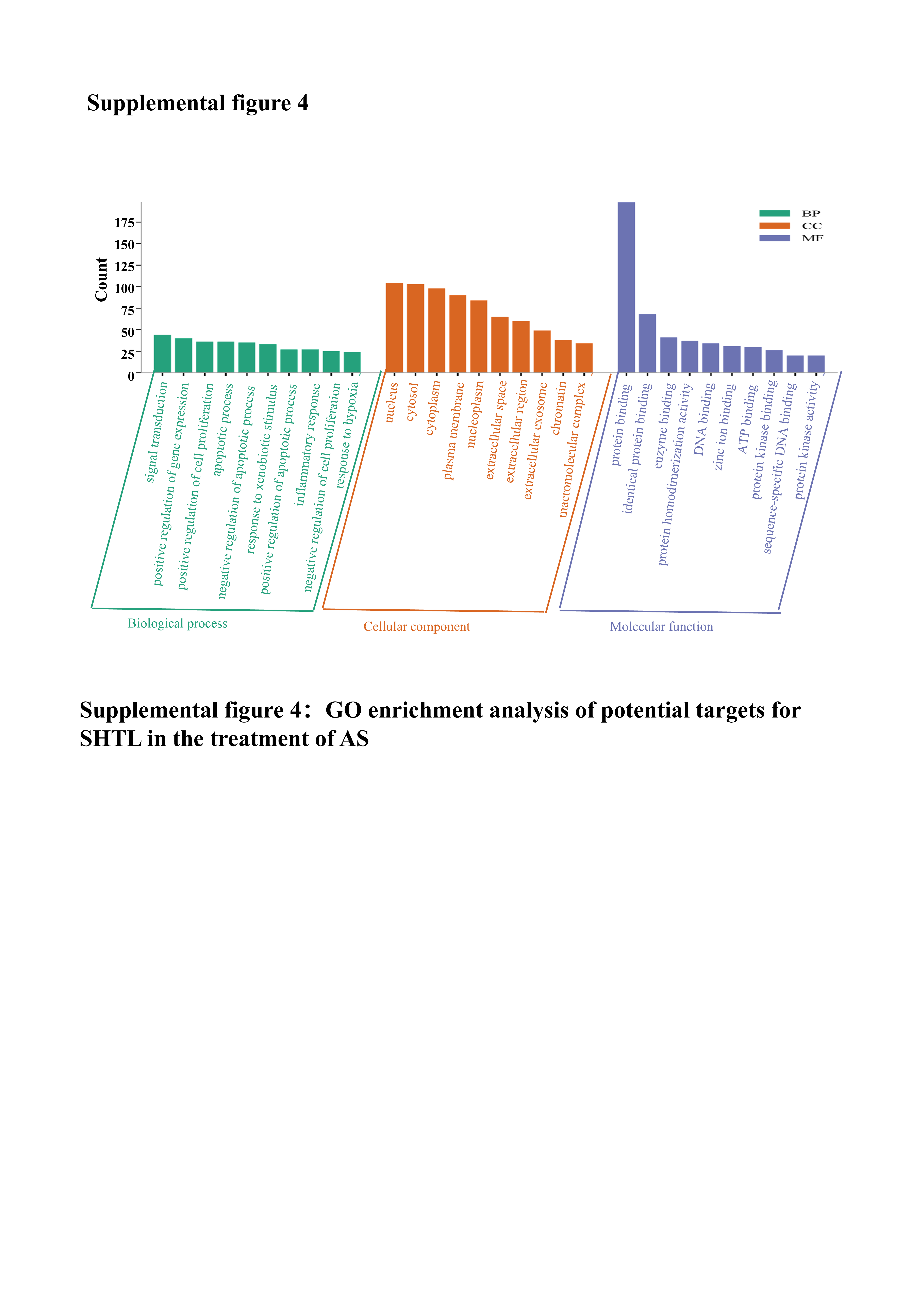

Supplement: Supplementary file 6 [file Image4.tif]

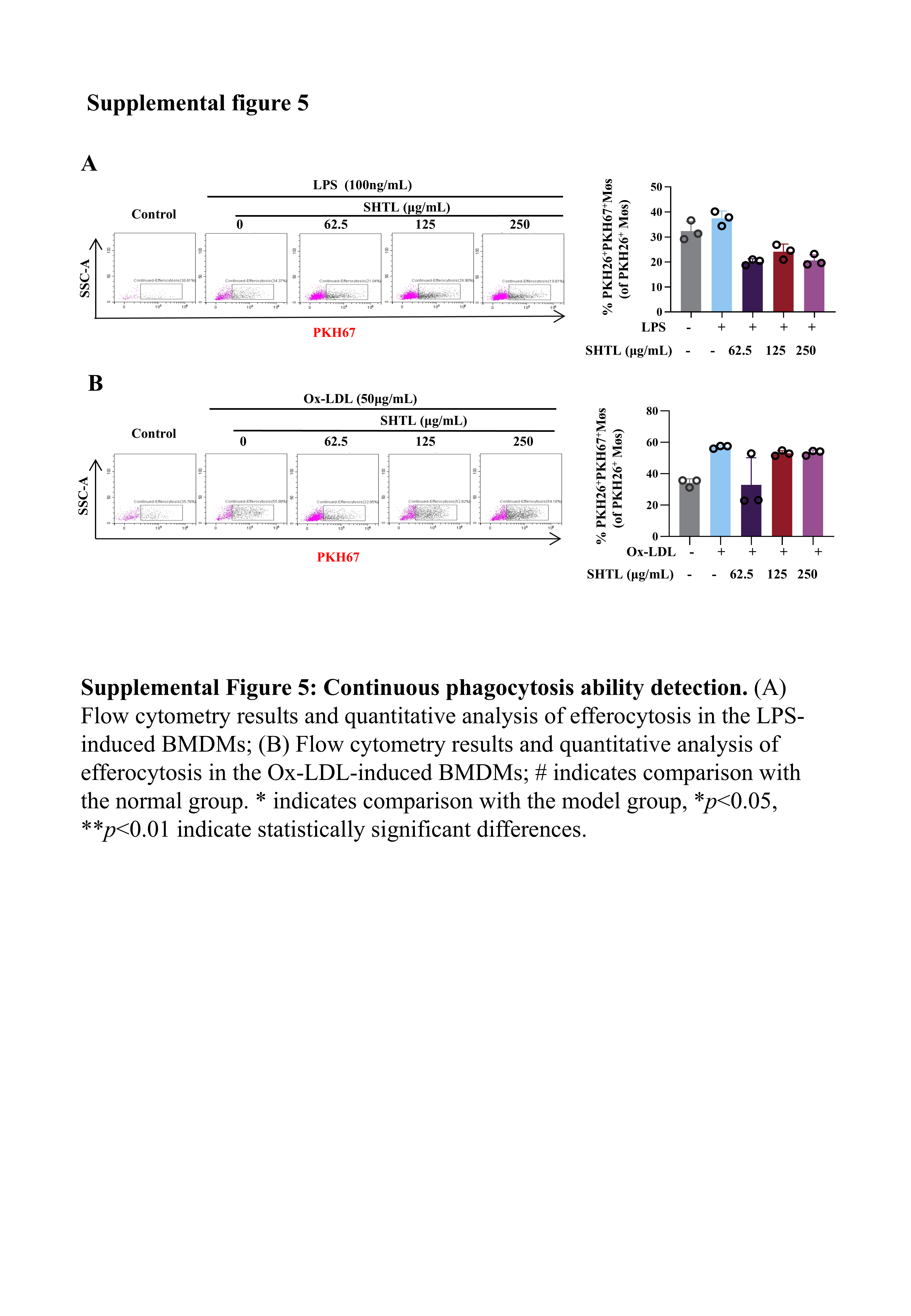

Supplement: Supplementary file 7 [file Image5.tif]

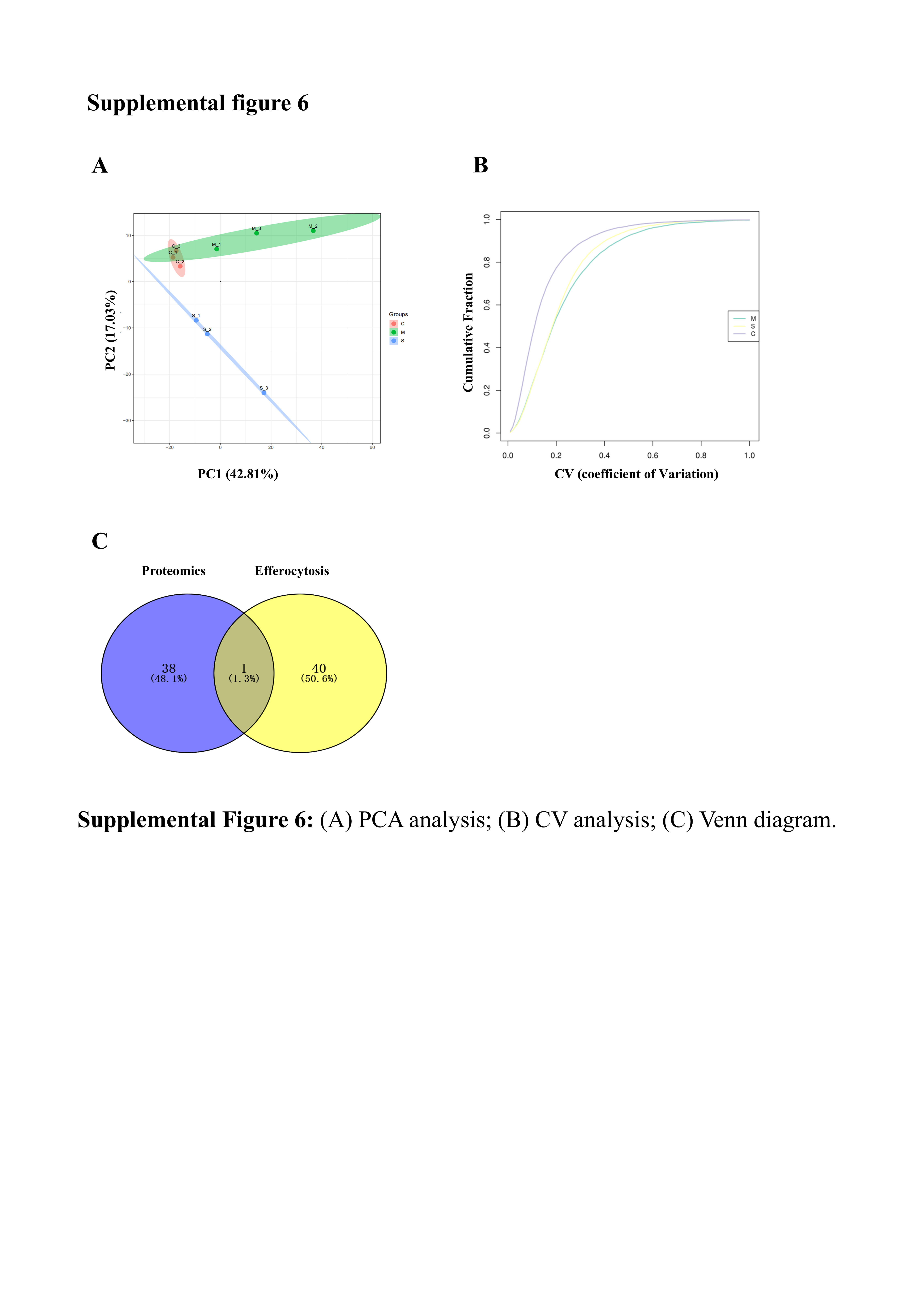

Supplement: Supplementary file 8 [file Image6.tif]

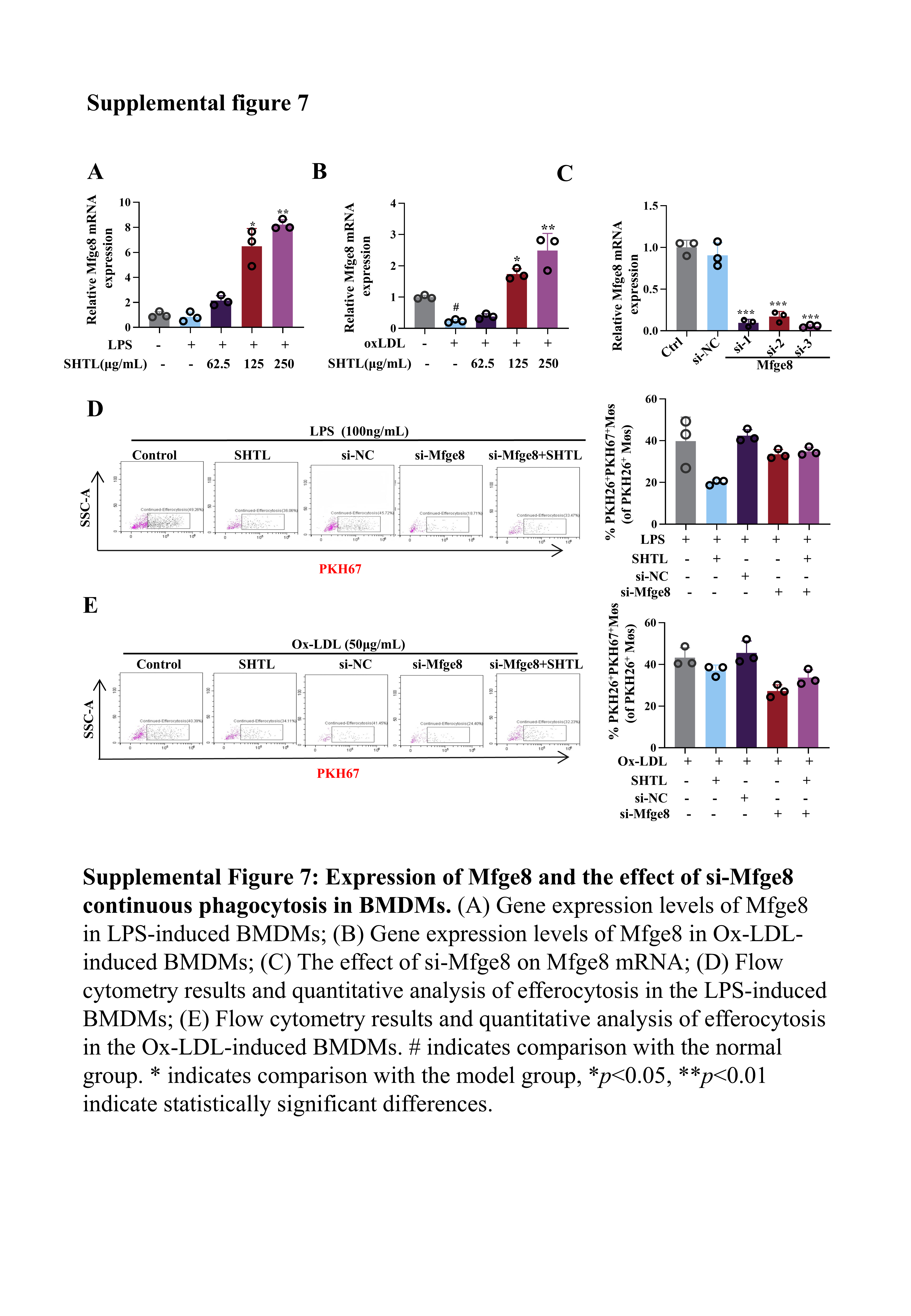

Supplement: Supplementary file 9 [file Image7.tif]

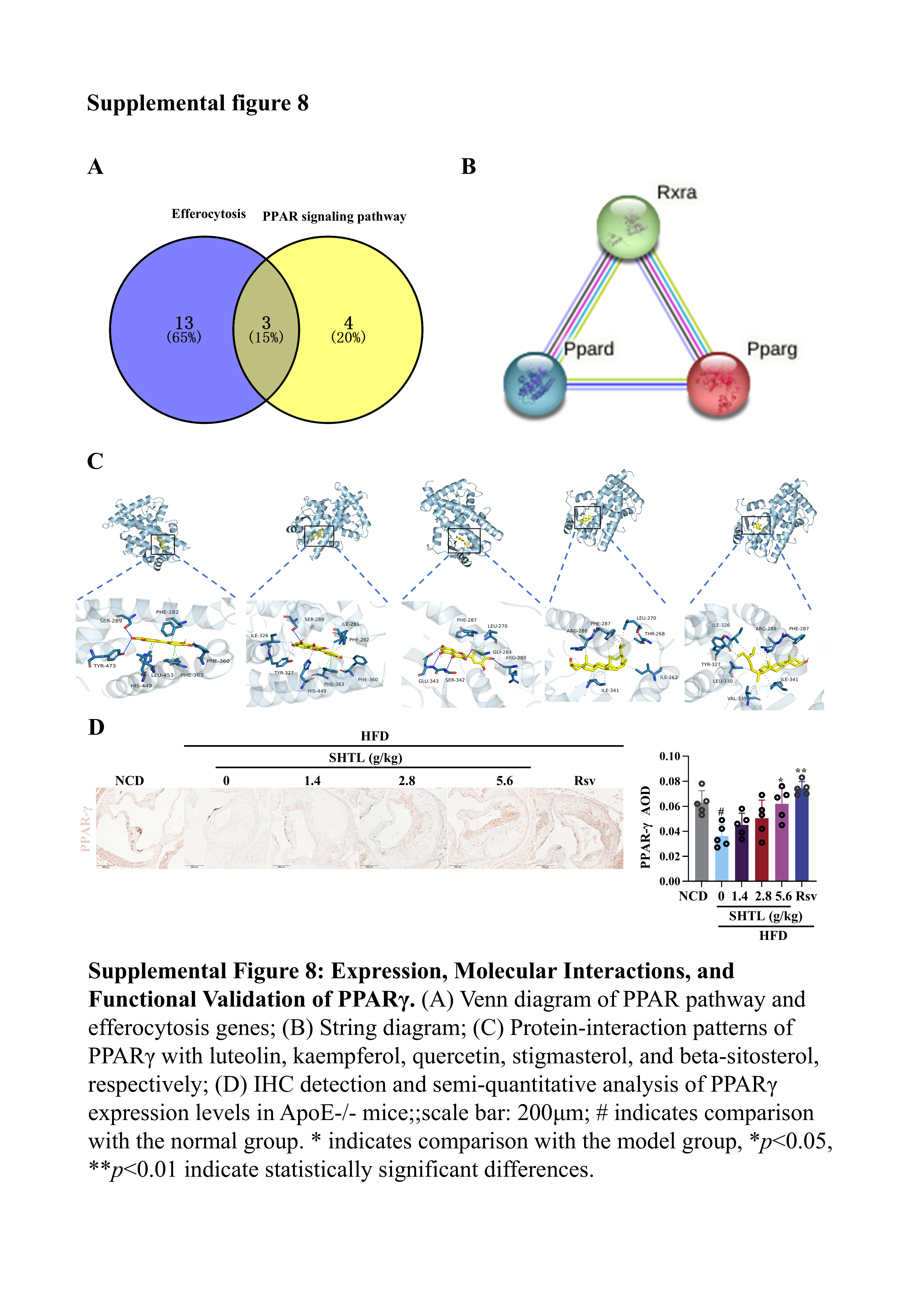

Supplement: Supplementary file 10 [file Image8.tif]

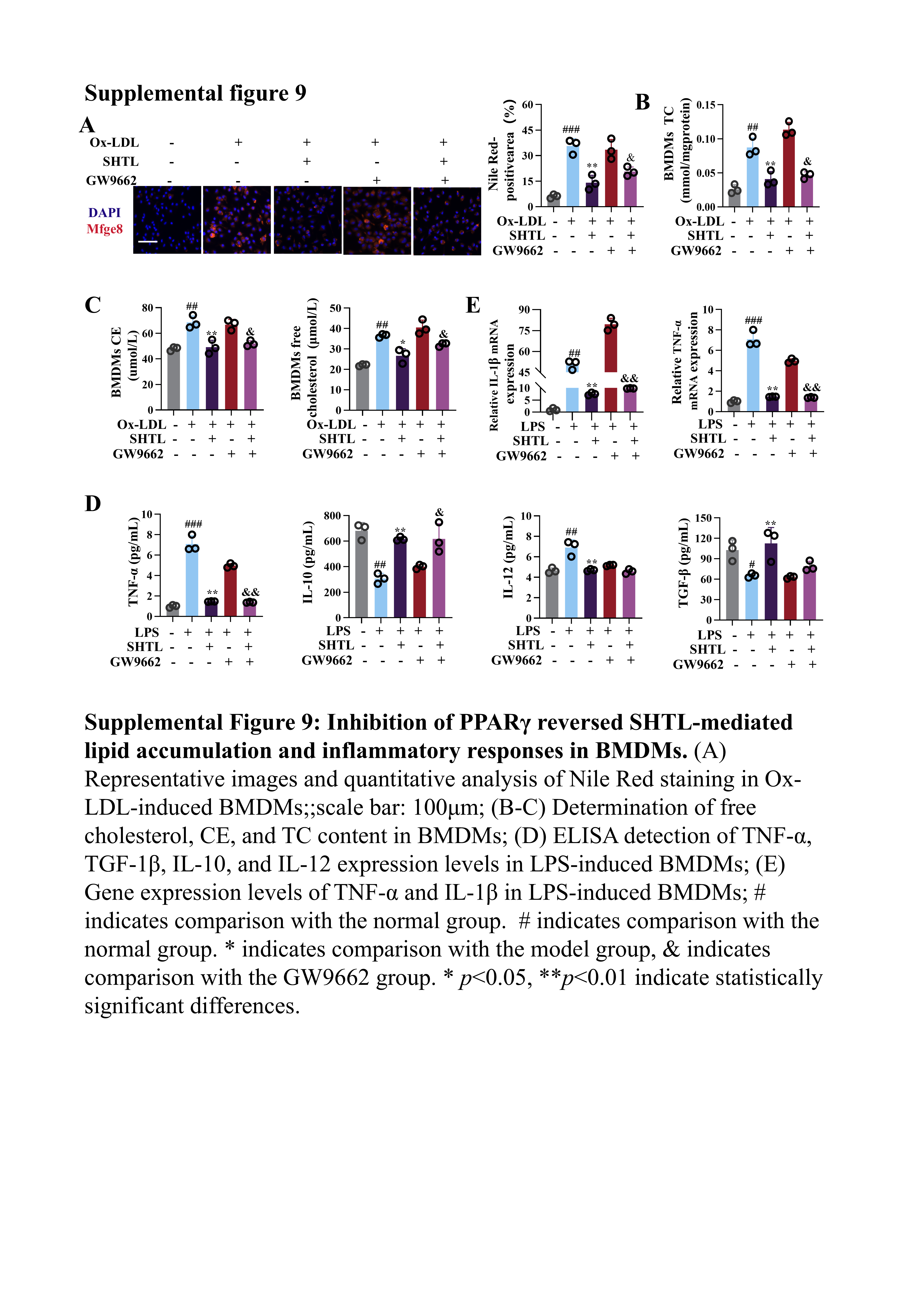

Supplement: Supplementary file 11 [file Image9.tif]

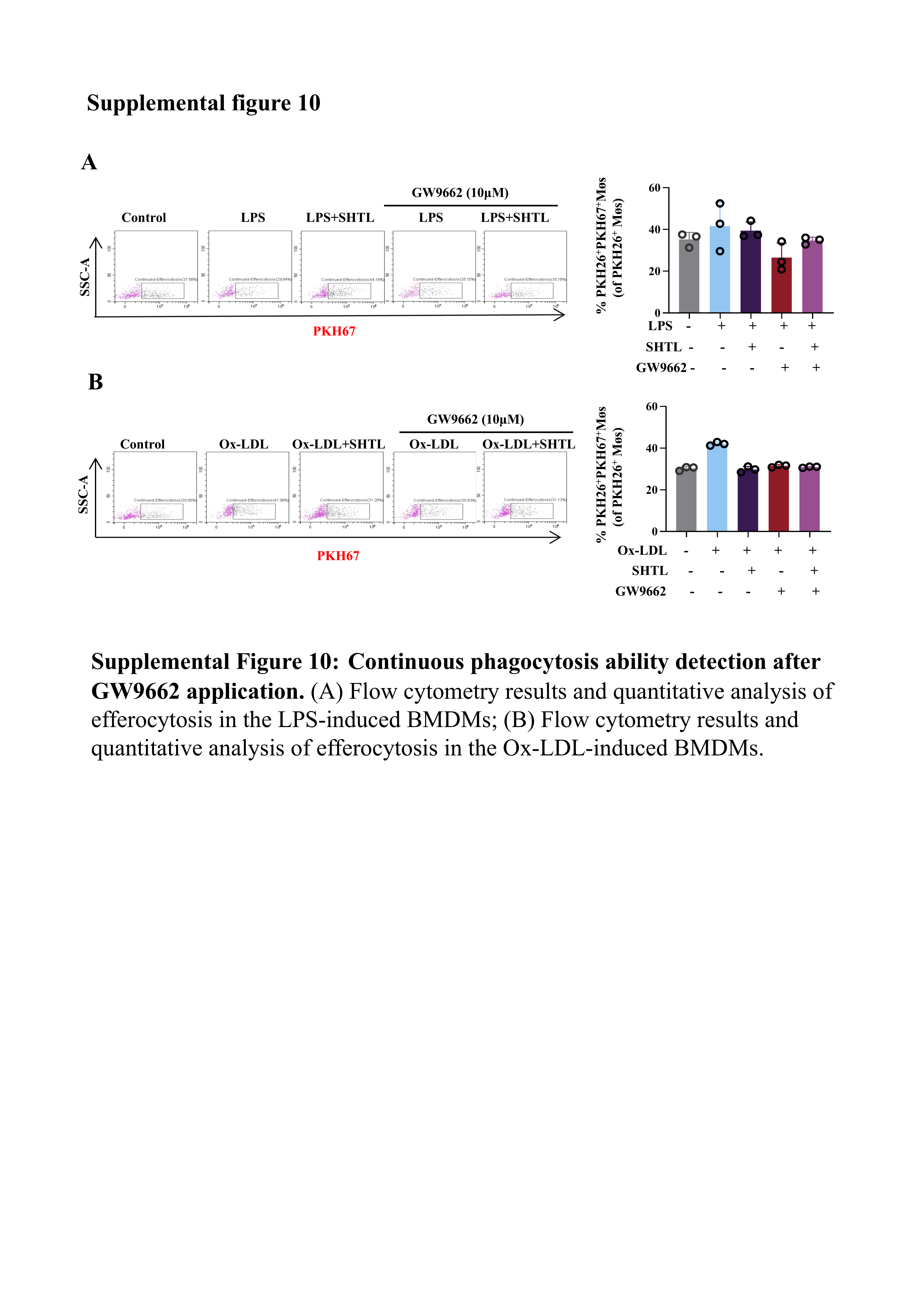

Supplement: Supplementary file 12 [file Image10.tif]

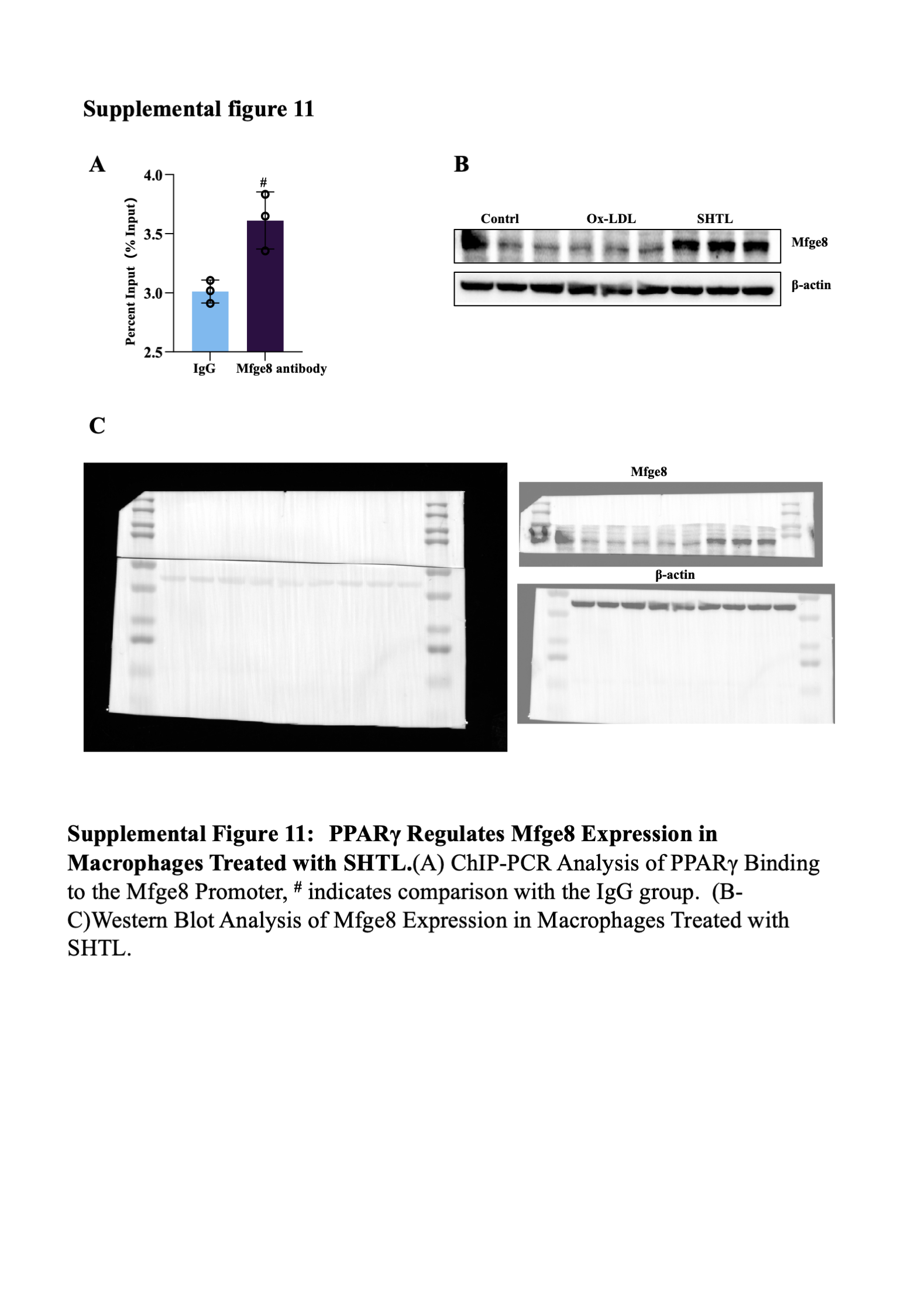

Supplement: Supplementary file 13 [file Image11.tiff]
