# Supplementary material for: Shen-Hong-Tong-Luo formula ameliorates atherosclerosis by enhancing macrophage efferocytosis through activating the PPARγ/mfge8 pathway
Source: Front Immunol. 2026 Jan 20;16:1727378. doi: 10.3389/fimmu.2025.1727378 (PMC12864095; doi:10.3389/fimmu.2025.1727378)
Supplement: Supplementary file 14 [file Table1.docx]

Supplemental Table 1 The compositions of the SHTL formula

| Chinese name | Latin name | Family | Weight (g) | Part used |
| --- | --- | --- | --- | --- |
| Ren shen | Panax ginseng C.A. Mey | Araliaceae | 20 | Root |
| Dan shen | Salvia miltiorrhiza Bunge | Lamiaceae | 25 | Root |
| Hong jingtian | Rhodiola crenulata | Crassulaceae | 15 | Root |
| Jin yinhua | Lonicera japonica Thunb | Caprifoliaceae | 15 | Flower |
| Chi shao | Paeonia anomala subsp. veitchii (Lynch) D.Y.Hong & K.Y.Pan | Ranunculaceae | 15 | Root |
| Gua lou | Trichosanthes kirilowii Maxim | Cucurbitaceae | 20 | Fruit |
| Dang gui | Angelica sinensis (Oliv.) Diels | Apiaceae | 20 | Root |
| Jiang xiang | Dalbergia odorifera T.C.Chen | Leguminosae | 10 | Rhizome |

Supplemental Table 2 PPAR γ docking results with core components

| target | SHTL compounds | Bindingenergy  (Kcal/mol) |
| --- | --- | --- |
| PPARγ | luteolin | -9.1 |
|  | quercetin | -8.1 |
|  | beta-sitosterol | -7.3 |
|  | kaempferol | -9 |
|  | Stigmasterol | -7.7 |
